# MDA5 Induces a Stronger Interferon Response than RIG-I to GCRV Infection through a Mechanism Involving the Phosphorylation and Dimerization of IRF3 and IRF7 in CIK Cells

**DOI:** 10.3389/fimmu.2017.00189

**Published:** 2017-02-24

**Authors:** Quanyuan Wan, Chunrong Yang, Youliang Rao, Zhiwei Liao, Jianguo Su

**Affiliations:** ^1^College of Fisheries, Huazhong Agricultural University, Wuhan, China; ^2^College of Veterinary Medicine, Huazhong Agricultural University, Wuhan, China

**Keywords:** grass carp (*Ctenopharyngodon idella*), grass carp reovirus, RIG-I, MDA5, IRF3, IRF7, IFN

## Abstract

Retinoic acid-inducible gene I (RIG-I) and melanoma differentiation-associated gene 5 (MDA5) are critical cytosolic sensors that trigger the production of interferons (IFNs). Though their recognition functions are well identified, their unique roles in the downstream signal transduction remain to be elucidated. Herein, we report the differential effect between grass carp (*Ctenopharyngodon idella*) MDA5 (CiMDA5) and CiRIG-I on the production of various IFNs upon grass carp reovirus (GCRV) infection in *C. idella* kidney (CIK) cell line. In CIK cells, grass carp IFN1 (CiIFN1) and CiIFN3 are relatively highly expressed while CiIFN2 and CiIFN4 are relatively slightly expressed. Following GCRV infection, CiMDA5 induces a more extensive type I IFN response than CiRIG-I. Further investigation reveals that both CiMDA5 and CiRIG-I facilitate the expression and total phosphorylation levels of grass carp IFN regulatory factor (IRF) 3 (CiIRF3) and CiIRF7 upon GCRV infection or poly(I:C) stimulation. However, the difference is that CiRIG-I decreases the threonine phosphorylation level of CiIRF7. As a consequence, CiMDA5 enhances the heterodimerization of CiIRF3 and CiIRF7 and homodimerization of CiIRF7, whereas CiRIG-I facilitates the heterodimerization but attenuates homodimerization of CiIRF7. Moreover, the present study suggests that CiIRF3 and CiIRF7 heterodimers and CiIRF7 homodimers are able to induce more extensive IFN-I responses than CiIRF3 homodimers under GCRV infection. Additionally, CiMDA5 induces a stronger type II IFN (IFN-II) response against GCRV infection than CiRIG-I. Collectively, these results demonstrate that CiMDA5 plays a more potent role than CiRIG-I in IFN response to GCRV infection through differentially regulating the phosphorylation and dimerization of CiIRF3 and CiIRF7.

## Highlights

1. CiMDA5 and CiRIG-I increase the phosphorylation levels rather than the mRNA and protein expressions of CiIRF3 and CiIRF7.
2. CiMDA5 enhances the heterodimerization of CiIRF3 and CiIRF7 and homodimerization of CiIRF7, whereas CiRIG-I facilitates the heterodimerization but attenuates the homodimerization of CiIRF7.
3. CiMDA5 induces a stronger IFN response against GCRV infection than CiRIG-I.

## Introduction

Vertebrates are armed with innate and adaptive immune systems to withstand invasive viruses and other microbes. The interferon (IFN)-mediated innate immune response is the first line of defense against various pathogens (1). In which, pattern recognition receptors that detect the conserved patterns or structures, including bacterial cytoderm components, non-self nucleic acids, and certain highly conserved proteins, play a vital role in initiating IFN signaling pathways. The retinoic acid-inducible gene I (RIG-I)-like receptors (RLRs), including RIG-I, melanoma differentiation-associated protein 5 (MDA5), and laboratory of genetics and physiology 2 (LGP2), are crucial PRRs in the recognition of viral RNA in the cytosol (2). They share a DExD/H-box RNA helicase domain that hydrolyzes ATP to unwind RNA and a C-terminal autoregulatory domain (CTD or RD) that is responsible for initial RNA binding (3). Another pivotal domain, i.e., tandem N-terminal caspase recruitment domains (CARDs) that exist in MDA5 and RIG-I but not in LGP2 are the essential components in the signal transduction. Upon binding with ligands, MDA5 and RIG-I undergo reconfiguration to release the RD-repressed CARDs, which then recruit and interact with the CARD in mitochondrion-adherent adaptor named as mitochondrial antiviral signaling protein (MAVS, also known as IPS-1, VISA, and Cardif) (4). Once the CARD–CARD interaction shapes, MAVS forms functional prion-like aggregates and recruits several tumor receptor-associated factors (TRAFs) to activate IκB kinases (IKKs) and TRAF-associated NF-κB activator-binding kinase (TBK) 1 (5, 6), and thereby recruits IFN regulatory factor (IRF) 3 for its phosphorylation activation and the subsequent nucleus translocation (7). In this regard, another major transcription factor IRF7, a typical IFN-stimulated gene (ISG), is implicated in RLR signaling pathway as well (8, 9). Similar to IRF3, phosphorylation-activated IRF7 undergoes nucleus translocation and then cooperates with activated IRF3 to bind the promoter regions of IFN genes for the expression initiation following viral infection (10).

As key cytokines in the innate immune response, IFNs exhibit various biological functions, including antiviral activity, antitumor activity and immunomodulatory effects (11). In mammals, most of the IFN family members are well characterized and classified into three types, namely type I (IFN-I), II (IFN-II), and III (IFN-III), according to their structures and receptor complexes (1, 11). Since IFNs are vital and complicated players in antiviral immunity, much attention has been paid on their evolution characterization, which gives rise to broad identification studies on fish IFNs (12–14). As the two excellent review literature studies summarized, teleost fishes possess IFN-I and IFN-II, and IFN-I is further classified into group I and II according to the number of cysteines they contained (15, 16). In zebrafish (*Danio rerio*), group I IFN-I includes IFN1 and IFN4, while group II IFN-I includes IFN2 and IFN3, and IFN-II contains two members: IFNγ1 and IFNγ2 (16). Each kind of IFN performs its own functions: group I IFN-I is responsible for inducting most of the ISGs, while group II IFN-I as rapid and transient agonist antiviral genes serves as a complement of group I IFN-I (17), and IFN-II contributes to the phagocytic and nitric oxide responses of phagocytes and the regulation of some cytokines and chemokines (16).

Another member of RLRs, LGP2 does not possess a CARD, which leads to an incessant controversy about its functions in antiviral immunity (18–20). Although the function of LGP2 and the shared functions of MDA5 and RIG-I are subjects of great interest to immunologist, the different roles of MDA5 and RIG-I should be paid more attention as well, which may contribute to better understanding of the fine regulation of RLRs. At present, overwhelming reports have focused on the similar and different roles of MDA5 and RIG-I in the recognition of pathogens (21–23), which state that MDA5 and RIG-I play a complementary and non-redundant role in the pattern recognition. However, it is interesting that RIG-I cannot be identified in some fishes, such as Japanese pufferfish (*Takifugu rubripes*), medaka (*Oryzias latipes*), stickleback (*Gasterosteus aculeatus*), and large yellow croaker (*Pseudosciaena crocea*) (24–26), which arouses an interest in the investigation on the shared and unique roles of fish MDA5 and RIG-I in the downstream signal transduction. In addition, considering the complexity of fish IFNs in antiviral immunity, it is of great interest to investigate the different IFNs induced by fish MDA5 and RIG-I.

Previously, we attested the critical roles of full-length MDA5 and RIG-I and their domains in antiviral immunity in grass carp (*Ctenopharyngodon idella*) (27–30), but the differential roles of MDA5 and RIG-I were not well characterized. And recently, the IFN system in grass carp was clarified, which suggests that there are six grass carp IFNs (CiIFNs): CiIFN1 and CiIFN4 belong to group I IFN-I; CiIFN2 and CiIFN3 belong to group II IFN-I; while CiIFNγ1 and CiIFNγ2 belong to IFN-II (31). Based on these available data, the present study reveals that *C. idella* MDA5 (CiMDA5) and CiRIG-I differentially induce the production of IFNs. Further investigations show that CiMDA5 and CiRIG-I facilitate the protein phosphorylation rather than mRNA and protein expression levels of *C. idella* IRF3 (CiIRF3) and CiIRF7 and give rise to different dimerization forms of CiIRF3 and CiIRF7 under immunostimulation. Our findings demonstrate that CiMDA5 plays a more potent role in the pathway of IFN induction than CiRIG-I in *C. idella* kidney (CIK) cells.

## Materials and Methods

### Cell Culture, Virus, and Reagents

*C. idella* kidney cells were provided by China Center for Type Culture Collection. Fathead minnow cell line (FHM) was a kind gift from Dr. Junfa Yuan, Huazhong Agricultural University, Wuhan, China. Previous established overexpression cells, i.e., stably transfected MDA5 (MDA5+), RIG-I (RIG-I+), and enhanced GFP (EGFP+) cells, were renewedly cultured (27, 30). Cells were grown in DMEM (Gibco) supplemented with 10% FBS (Gibco), 100 U/ml penicillin, and 100 U/ml streptomycin and maintained at 28°C in a humidified atmosphere of 5% CO_2_ incubator (Thermo Scientific). Geneticin (G418) (200 μg/ml) (Sigma-Aldrich) was added to maintain MDA5+, RIG-I+, and EGFP+ cells. Grass carp reovirus (GCRV) was propagated in CIK cells and stored at −80°C.

Polyinosinic:polycytidylic acid [poly(I:C)], isopropyl-d-1-thiogalactopyranoside (IPTG), serine/threonine phosphatase inhibitor, tyrosine phosphatase inhibitor, and protease inhibitor cocktails were purchased from Sigma-Aldrich. Hoechst 33342 was from AAT Bioquest. FuGENE^®^ 6 transfection reagent was purchased from Promega. Calf intestinal alkaline phosphatase (CIP) was purchased from NEB. All the restriction enzymes were purchased from Thermo Scientific. Lysis buffer [20 mM Tris (pH 7.5), 150 mM NaCl, 1% Triton X-100, and a handful of compounds containing sodium pyrophosphate, β-glycerophosphate, sodium orthovanadate, sodium fluoride, EDTA, and leupeptin] was purchased from Beyotime, Shanghai, China. Nuclear and cytoplasmic protein extraction kit was purchased from Beyotime. All the primer synthesis and DNA sequencing were carried out in AuGCT biotechnology Co., Ltd., Wuhan, China.

### Expression Vectors/Recombinant Plasmids

The whole open reading frames of CiIRF3 gene (GenBank accession no. KC898261) and CiIRF7 gene (GenBank accession no. GQ141741) were amplified from cDNA derived from head kidney tissue of grass carp. Then the CiIRF3 or CiIRF7 overexpression vectors (pIRF3 or pIRF7) and tag-labeled vectors (pIRF3-Flag, pIRF3-*myc*, pIRF7-Flag, and pIRF7-*myc*) were singly constructed by insertion of the corresponding PCR amplicons into the *Eco*RI/*Kpn*I sites of pdCMV vector (Figure S1A in Supplementary Material) as described in our previous report (27). For subcellular localization experiment, EGFP-fused vectors of CiIRF3 (or CiIRF7), i.e., pIRF3-EGFP and pIRF7-EGFP were constructed by insertion of the PCR amplicons into *Kpn*I/*Bam*HI and *Kpn*I/*Apa*I sites of psCMV (Figure S1B in Supplementary Material). The 5′-flanking sequences of CiIRF3, CiIRF7, CiIFN3, and CiIFN4 genes were obtained from the genome data of grass carp (32) and confirmed using Sanger sequencing (ABI 3730 DNA Analyzer). The promoter regions of them were predicted using PROSCAN program (version 1.7) (33). For promoter identification, pIRF3pro-EGFP, pIRF7pro-EGFP, pIFN3pro-EGFP, and pIFN4pro-EGFP plasmids were singly constructed by substituting the CMV promoter with either the 516 bp fragment of CiIRF3, 901 bp fragment of CiIRF7, 1,023 bp fragment of CiIFN3, or 1,610 bp fragment of CiIFN4, which contained the corresponding predicted promoter region in the *Xho*I/*Hin*dIII sites of psCMV. Subsequently, the validated 5′-flanking sequences were singly inserted into the *Xho*I/*Hin*dIII sites of pGL3-basic luciferase reporter vector (Promega), and the constructed plasmids were named as pIRF3pro-Luc, pIRF7pro-Luc, pIFN3pro-Luc, and pIFN4pro-Luc, respectively. The primers used for constructs are listed in Table S1 in Supplementary Material, and all the PCR amplicons were validated by Sanger sequencing. Additionally, pMDA5-HA, pRIG-I-HA, pIFN1pro-Luc, pIFN2pro-Luc, pIFNγ1pro-Luc, and pIFNγ2pro-Luc plasmids were constructed before in our laboratory.

### Transfection, Infection, Confocal Fluorescence Microscopy, and Luciferase Activity Assay

To establish stably overexpressed and EGFP fusion-expressed cells, 0.8 μg of either pIRF3, pIRF7, pIRF3-EGFP, or pIRF7-EGFP vector was transfected into CIK cells, respectively, as previous description in detail (34). Meanwhile, pIRF3pro-EGFP and pIRF4pro-EGFP were transfected into CIK cells as well, and the expression of EGFP was assessed by imaging with a fluorescence microscope (Leica). For GCRV infection or poly(I:C) stimulation, cells were equally aliquoted into 12-well or 6-well plates in advance. After washing the monolayer cells thrice with fresh serum-free DMEM, serum-free DMEM with GCRV [multiplicity of infection (MOI) = 1] or poly(I:C) (final concentration is 5 μg/ml) was added into the wells of experiment group, while serum-free DMEM with commensurate phosphate-buffered saline (PBS) was added into the wells of control group. For confocal fluorescence microscopy, pIRF3-EGFP and pIRF7-EGFP stably transfected cells were equably seeded on microscope coverslips (Fisher Scientific) in 12-well plates for 24 h, then washed with fresh DMEM for either GCRV infection, poly(I:C) stimulation or PBS treatment (control). Twenty-four hours later, those cells were washed thrice with PBS and fixed with 4% (v/v) paraformaldehyde for 15 min at room temperature. For nuclear staining, cells were incubated in 0.1 mg/ml Hoechst 33342 for 10 min in a darkroom. The observation of subcellular location was performed using an UltraVIEW VoX 3D Live Cell Imaging System (PerkinElmer).

For luciferase reporter assays, either CIK or FHM cells were seeded in 24-well plates overnight, followed by being co-transfected with the overexpressed plasmid, target promoter-luciferase plasmid, and pRL-TK (internal control reporter vector) at a ratio of 10:10:1 using FuGENE^®^ 6 transfection reagents (Promega). Simultaneously, pdCMV, objective promoter-luciferase plasmid, and pRL-TK vectors were co-transfected as vehicle control. Luciferase activities were measured using the Dual-Luciferase Reporter Assay System (Promega) and a VICTOR™ X Series Multilabel Plate Reader (PerkinElmer). Data were normalized to the amounts of Renilla luciferase activities according to the protocol.

### Preparations of Polyclonal Antisera and Commercial Antibodies

For the acquisition of anti-IRF7 polyclonal antiserum, the full-length coding sequence of CiIRF7 gene was cloned into *Eco*RI/*Xho*I sites of pET-32a(+) vector (Novagen) for prokaryotic expression. The plasmid pET-32a(+)-IRF7 was transformed into the *Escherichia coli* BL21(DE3) pLysS cells (Novagen). A single isolated colony of transformant was inoculated in 5 ml of LB medium containing 100 μg/ml ampicillin and incubated for 12 h. The overnight culture was diluted 1:100 in 400 ml of LB medium containing same antibiotics. The culture was grown to an A600 of 0.6 and induced by addition of 1 mM IPTG. The bacteria were harvested after 5 h induction. The recombinant protein was extracted according to the classical protocol (35) and purified using Ni-IDA-Sefinose™ Resin Kit (Sangon Biotech, Shanghai, China). The purified protein was confirmed by Western blotting with Anti-His tag mouse monoclonal primary antibody (1:2000) (Abbkine), and then applied to immunize New Zealand white rabbits to acquire the polyclonal anti-IRF7 antiserum according to the protocols mentioned in previous reports (36). As a kind gift, anti-IRF3 rabbit polyclonal antiserum was previously prepared by Professor Yibing Zhang, Institute of Hydrobiology, Chinese Academy of Sciences, Wuhan, China (37). Phosphoserine, phosphothreonine, and phosphotyrosine antibodies were purchased from ImmuneChem. Anti-Flag tag (ab45766), anti-HA tag (ab18181), anti-*myc* tag (ab32) mouse monoclonal antibodies, and anti-β-tubulin primary rabbit polyclonal antibody (ab6046) were purchased from Abcam. Anti-H3 primary rabbit polyclonal antibody was purchased from Beyotime. IRDye^®^ 800CW Donkey anti-rabbit-IgG and anti-mouse-IgG (H+L) secondary antibodies were purchased from LI-COR.

### Western Blotting and Immunoprecipitation (IP)

For Western blotting analysis, cells were plated in 6-well plates, incubated overnight, and subsequently treated with PBS, GCRV, or poly(I:C). At 24 h posttreatment, cells were washed with PBS and lysed in lysis buffer supplemented with 1 mM phenylmethylsulfonyl fluoride, serine/threonine phosphatase inhibitor, tyrosine phosphatase inhibitor, and protease inhibitor cocktails. After clarification by centrifugation at 12,000 rpm for 15 min, 30 μg of supernatant proteins was separated by 8–12% SDS-PAGE. The separated polypeptides were electroblotted onto nitrocellulose (NC) filter membranes (Millipore) using a trans-blot SD semidry electrophoretic transfer cell (Jim-X, Dalian, China), and then the blotted membranes were incubated with blocking TBST buffer (0.5 M Tris-Cl, 150 mM NaCl, 0.5% Tween 20, and 1% bovine serum albumin) at room temperature for 2 h or 4°C overnight. Subsequently, the membranes were incubated with appropriate primary antibodies for 2 h at room temperature or overnight at 4°C in blocking TBST buffer, washed thrice with TBST buffer, and then incubated with secondary antibody for 1 h at room temperature. After again washing thrice with TBST buffer, the NC membranes were scanned and imaged by an Odyssey^®^ CLx Imaging System (LI-COR). For hybridization, the anti-IRF3 and anti-IRF7 antisera were diluted 1:1,000, commercial primary antibodies 1:5,000, and secondary antibodies 1:10,000.

To determine the phosphorylation and dimerization status of CiIRF3 and CiIRF7, IP and Co-IP were performed. Whole-cell lysates were prepared in the presence of abovementioned lysis buffer, and the cellular debris was removed by centrifugation at 12,000 rpm for 30 min at 4°C. The supernatant was transferred to a fresh tube and incubated with 1 μg antibodies overnight at 4°C, followed by incubation with 30 μl protein A+G sepharose beads (Beyotime) for 2 h at 4°C. Then the beads were washed with lysis buffer four times and eluted with 20 μl 2 × SDS loading buffer by boiling for 10 min. The precipitates were detected by immunoblotting with the corresponding antibodies.

### Viability Tests of CiIRF3 and CiIRF7 Overexpressed Cells upon GCRV Infection

*C. idella* kidney cells that overexpressed either CiIRF3, CiIRF7, or EGFP were severally seeded in 96-well plates (1 × 10^4^ cells/well). After being incubated overnight, the cells were infected with GCRV. At the scheduled time, 20 μl of 3-(4,5-dimethylthiazol-2-yl)-2,5-dimethyltetrazolium bromide (5 mg/ml in PBS) was added to each well. After 4 h of incubation at 28°C, DMSO (100 μl/well) was added at 28°C for 10 min. The OD was measured by a microplate reader (Infinite F200, Tecan) at 490 nm. Data were expressed as viability index, which is the ratio of the mean OD value of quartic wells measured at corresponding time point to the mean value of quartic wells measured at 0 h postinfection (p.i.).

### Semi-Quantitative and Real-time Quantitative RT-PCR (RT-qPCR)

Total cellular RNA isolation and cDNA synthesis were performed according to the protocol described in the previous reports (38). For semiquantitative RT-PCR, 200 ng of cDNA for each target gene amplification was used according to the following procedure: 5 min predenaturation at 94°C, amplification and extension for 35 cycles (20 cycles for internal control) at 94°C for 30 s, 60°C for 30 s, and 72°C for 20 s. The amplicons were analyzed by agarose electrophoresis and imaged using Gel Doc XR system (Bio-Rad). For RT-qPCR, the mRNAs of target genes were quantified using SYBR Premix Ex Taq II reagent (Takara) and a LightCycler 480 II Real-time PCR system (Roche). The PCR reactions were cycled during the real-time detection. Primers are listed in Table 1. The mRNA expression levels were normalized to the expression level of EF1α, and the data were analyzed using the 2^–ΔΔCt^ method as described previously (39).

**Table 1:** **Primers for real-time quantitative RT-PCR analysis**.

Gene	Primer name	Forward primer (5′–3′)	Primer name	Reverse primer (5′–3′)
EF1α	EF125	CGCCAGTGTTGCCTTCGT	ER126	CGCTCAATCTTCCATCCCTT
Retinoic acid-inducible gene I	RF230	ACTACACTGAACACCTGCGGAA	RR231	GCATCTTTAGTGCGGGCG
Melanoma differentiation-associated gene	MF150	CAGGAGCGACTCTTGGACTATG	MR151	AAAGACGGTTTATTTGAATGGAAG
IFN regulatory factor 3 (IRF3)	IF960	ACTTCAGCAGTTTAGCATTCCC	IR961	GCAGCATCGTTCTTGTTGTCA
IRF7	IF767a	CGCCTGTGTTCGTCACTCGT	IR768a	GGTGGTTGGAAAGCGTATTGG
Interferon 1 (IFN1)	IF590	AAGCAACGAGTCTTTGAGCCT	IR591a	GCGTCCTGGAAATGACACCT
IFN2	IF439	TCTTTTTCCTCGTGAATGCTTG	IR433	TCACAACGATGTTCTGACTGGA
IFN3	IF435	TACATTTATAGAGACTGCGGGTGG	IR357	TGGAGTGTCTGGTAAACAGCCTT
IFN4	IF354	GTTCGTCATTCAGGCTCTGGTAG	IR436	TCCCTCCATCCTCCTTGTTCA
IFNγ1	IgF339	CGAGATGACCCATTTGGAGAC	IR390	CTTTGAAACCCATTCTGTGCC
IFNγ2	WF79	CAGCGAACACCTGAAACTAACA	WR80	CCATCCCAAAGTCATCAAACAT
IL-4	ILF559	GCACTGACATTTGTAGCCGTTA	ILR560	ATGGTTATGTAGGGTCTGGTTCA
IL-10	ILF561	TTGCCATTGTGACATTTTCCAG	ILR562	ATGATGACGTGAGTCGAGTTTGA
IL-12	ILF1702b	CTTTGTCGGGGTCCTAATTATGT	ILR1552	GTGCTTTTGCTTTGATGATGGA
GCRV-induced gene 1	GigF598	CTGCCCCTGCTGAAATGCT	GigR599	AGCCAAAGTTTCCATTCTGAGG
IFI56	IFIF596	TCTGGAGGGACTGAAGATTGGT	IFIR597	TGCGTTCGTTTCGTTCTTGTAG
ISG15	ISGF604	CCCCTTTCCAAGTGTTCGTC	ISGR605	ATGGTGCTTCCAGATGTGATGT
Mx2	MF428	ACATTGACATCGCCACCACT	MR429	TTCTGACCACCGTCTCCTCC

### Data Analysis

Statistical analysis and presentation graphics were carried out using Graphpad Prism 6.0 software. Unpaired Student’s *t*-test was used in the data analysis, and the *P* value <0.05 was considered as a statistically significant difference (*P < 0.05, **P < 0.01, ***P < 0.001).

## Results

### CiMDA5 and CiRIG-I Differentially Induce the Production of CiIFNs

**Figure 1:**
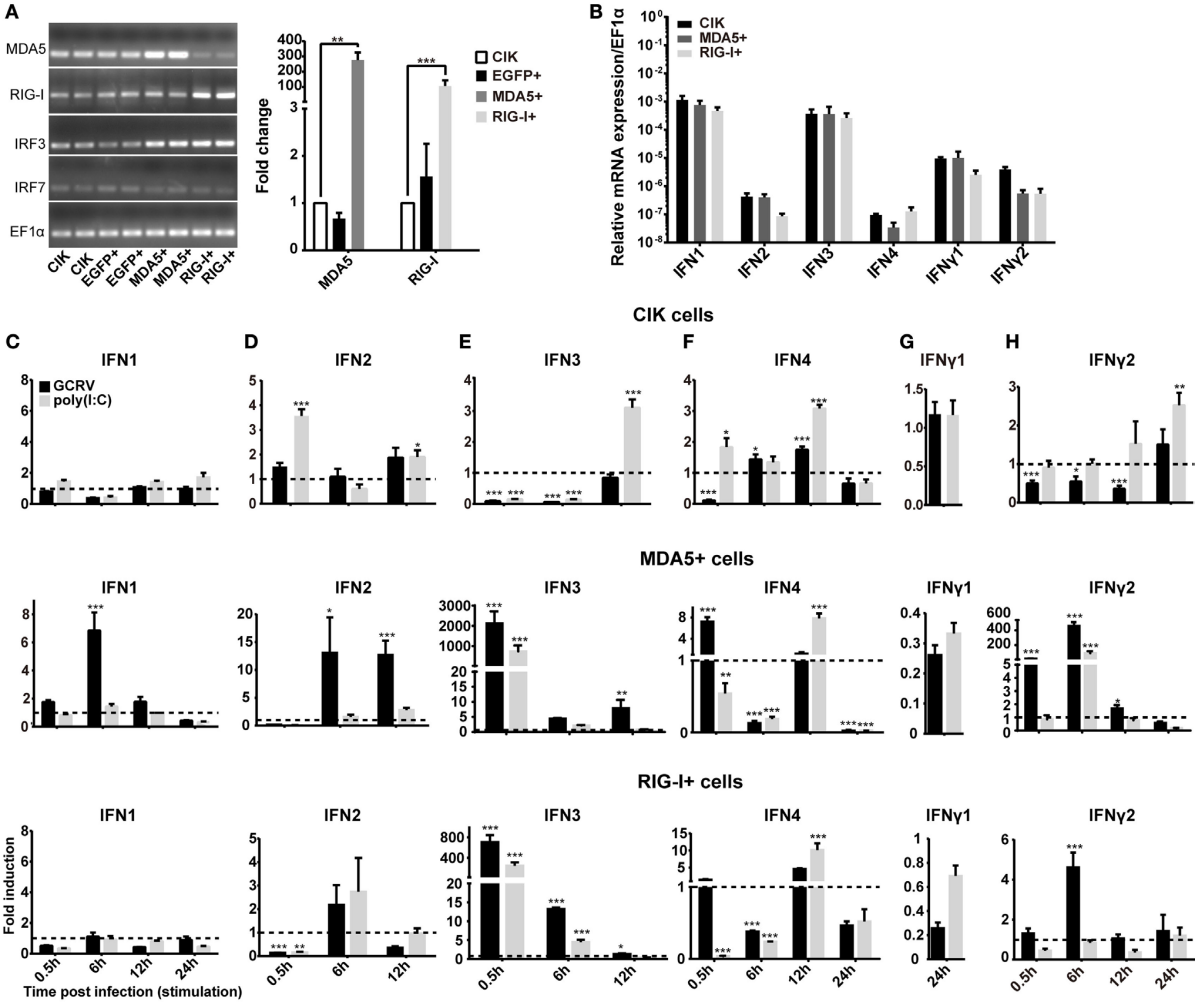
**Ctenopharyngodon idella melanoma differentiation-associated gene 5 (CiMDA5) and *C. idella* retinoic acid-inducible gene I (CiRIG-I) differentially induce the production of interferons (IFNs). (A)** EGFP+, MDA5+, and retinoic acid-inducible gene I (RIG-I)+ cells were seeded in 12-well plates for 24 h cultivation, and then total RNA samples were isolated from these cells for semiquantitative RT-PCR or RT-qPCR. **(B)** Total RNA samples isolated from *C. idella* kidney (CIK), MDA5+, and RIG-I+ cells were used to measure the relative expression levels of IFNs. Relative mRNA expression levels were normalized to *EF1*α in CIK cells. **(C–H)** CIK, MDA5+, and RIG-I+ cells were seeded in 12-well plates, and then were either phosphate-buffered saline (PBS) treated, grass carp reovirus (GCRV) infected, or polyinosinic:polycytidylic acid [poly(I:C)] stimulated. Total RNA samples were isolated at the scheduled time postchallenge. The relative expression levels of these genes were normalized by *EF1*α. Fold induction of gene expression level in CIK cells was obtained by comparing the normalized gene expression level in GCRV- or poly(I:C)-treated cells with that in PBS-treated cells (defined as 1) at the same time point, while those in MDA5+ and RIG-I+ cells were determined relative to corresponding treated CIK cells at the same time point. Data represent mean ± SEM of four independent wells of cells. *P < 0.05; **P < 0.01; ***P < 0.001.

To explore the differential role of CiMDA5 and CiRIG-I, MDA5+ and RIG-I+ cells were cultured, and the mRNA expression levels of CiMDA5 and CiRIG-I were detected. The result showed that CiMDA5 and CiRIG-I were overexpressed in MDA5+ and RIG-I+ cells, respectively (Figure 1A). Interestingly, it was unexpected that stable overexpressed CiRIG-I decreases the mRNA expression of endogenic CiMDA5, while CiMDA5 stable overexpression has no obvious effect on that of endogenic CiRIG-I. Next, we investigated the mRNA expression patterns of IFNs in CIK, MDA5+, and RIG-I+ cells. The result showed that CiIFN1 and CiIFN3 were the most abundant IFNs, followed by CiIFNγ1 and CiIFNγ2, while CiIFN2 and CiIFN4 were slightly expressed in CIK cells without immunostimulation (Figure 1B). Additionally, CiMDA5 and CiRIG-I were not able to affect the expression of CiIFNs without any immunostimulation. Subsequently, we asked whether CiMDA5 and CiRIG-I played an identical role in the induction of CiIFNs after immunostimulation. For sampling, a preexperiment was implemented, which indicated that 0.5 h, 6 h, 12 h, and 24 h were suitable for the detection of mRNA expression profiles of CiIFN1, CiIFN4, CiIFNγ1, and CiIFNγ2 and that 0.5 h, 6 h, and 12 h were suitable for that of CiIFN2 and CiIFN3. The following results showed that CiIFN1, CiIFN2 and CiIFN4 and CiIFNγ2 were slightly induced, while CiIFN3 was inhibited in GCRV-infected CIK cells at the early stage (0.5 h and 6 h p.i.) (Figures 1C–H, upper panels). Particularly, CiIFNγ1 could be detected just at 24 h p.i. or poststimulation, indicating that CiIFNγ1 is relatively insensitive to GCRV infection and poly(I:C) stimulation. These data suggest that the seldom expressed CiIFN2 and CiIFN4 play a positive role in GCRV infection or poly(I:C) stimulation in CIK cells.

**Figure 2:**
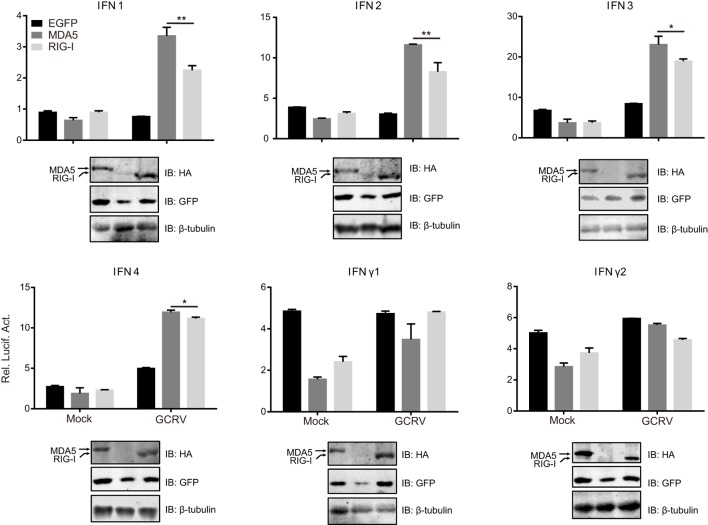
**Melanoma differentiation-associated gene 5 (MDA5) and retinoic acid-inducible gene I (RIG-I) differentially induce the promoter activities of various IFNs**. *Ctenopharyngodon idella* kidney cells seeded in 24-well plates were co-transfected with 380 ng pMDA5-HA or pRIG-I-HA, 380 ng pIFNpro-Luc (either pIFN1pro-Luc, pIFN2pro-Luc, pIFN3pro-Luc, pIFN4pro-Luc, pIFNγ1pro-Luc, or pIFNγ2pro-Luc), and 38 ng pRL-TK. Twenty-four hours later, one half of the cells were infected with grass carp reovirus, and the other half of the cells were treated with phosphate-buffered saline. Luciferase activities were detected at 6 h p.i. The remaining cell lysates were used for subsequent Western blotting tests to detect the overexpression of *C. idella* RIG-I-HA or CiMDA5-HA. Error bars represent ±SEMs obtained by testing each sample in quadruplicate. *P < 0.05; **P < 0.01.

To clarify the different roles of CiMDA5 and CiRIG-I in the induction of IFNs production, we tested the expression patterns of various IFNs in MDA5+, RIG-I+ and CIK cells by RT-qPCR. Relative to the expression levels in CIK cells, CiMDA5 overexpression strongly induced the expression of CiIFN1 at 6 h p.i., CiIFN2 at 6 h p.i. and 12 h p.i.; CiIFN3 and CiIFN4 at 0.5 h p.i.; CiIFNγ2 at 0.5 h p.i. and 6 h p.i., while inhibited CiIFNγ1 at 24 h p.i. under GCRV infection or poly(I:C) stimulation (Figures 1C–H, middle panels). Likewise, in CiRIG-I overexpressed cells infected with GCRV or stimulated with poly(I:C), the expression trend of each CiIFN could be described as that: CiIFN1 was not obviously affected; CiIFN2, CiIFN3, CiIFN4, and CiIFNγ2 was moderately induced at 6 h p.i., 0.5 h p.i., 12 h p.i., and 6 h p.i., respectively, while CiIFNγ1 was inhibited at 24 h p.i. (Figures 1C–H, lower panels). Subsequently, luciferase reporter assays were conducted to examine the effects of CiMDA5 and CiRIG-I on the promoter activities of CiIFNs. As shown in Figure 2, transient overexpressed CiMDA5-HA and CiRIG-I-HA did not affect the promoter activities of CiIFN-I at steady state, while enhanced the promoter activities of CiIFN-I under GCRV infection. Importantly, CiMDA5 induced a higher promoter activities than CiRIG-I. However, transient overexpressed CiMDA5-HA and CiRIG-I-HA decreased the promoter activities of CiIFN-II at steady state, while had no effects on them under GCRV infection. Furthermore, the expression levels of ISG, including GCRV-induced gene 1 (Gig1), IFN-induced gene 56 (IFI56), ISG15, and Mx2, were examined. As expected, CiMDA5 overexpression induced higher expression levels of CiGig1, CiIFI56, and CiMx2 than CiRIG-I overexpression under GCRV infection or poly(I:C) stimulation (Figure S2 in Supplementary Material). However, CiRIG-I and CiMDA5 overexpression did not have a significant induction effect on CiISG15 under immunostimulations. Collectively, these data illustrate that CiMDA5 induced a stronger IFN response than CiRIG-I in CIK cells under GCRV infection or poly(I:C) stimulation.

### CiMDA5 and CiRIG-I Have No Significant Effect on the Expression Levels of CiIRF3 and CiIRF7

**Figure 3:**
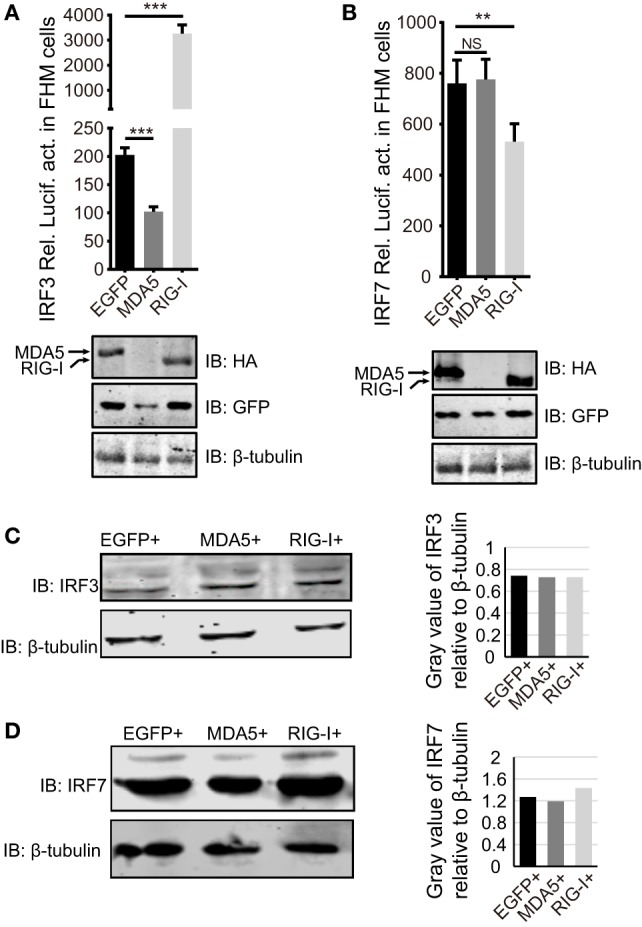
**Effect of *Ctenopharyngodon idella* retinoic acid-inducible gene I (CiRIG-I) and C. idella melanoma differentiation-associated gene 5 (CiMDA5) on the expression levels of C. idella IFN regulatory factor 3 (CiIRF3) and CiIRF7. (A,B)** Fathead minnow cells seeded in 24-well plates were co-transfected with 380 ng pRIG-I-HA (or pMDA5-HA or pCMV), 380 ng pIRF3pro-Luc (or pIRF7pro-Luc), and 38 ng pRL-TK. Twenty-four hours later, cells were harvested for detection of luciferase activity, and the remaining cell lysates were used for subsequent Western blotting tests to detect the overexpression of CiRIG-I-HA or CiMDA5-HA. **(C,D)** EGFP+, MDA5+, and RIG-I+ cells were seeded in 6-well plates for 24 h cultivation, and then the cell lysate was used for Western blotting analysis. The histograms beside the Western blotting results exhibit the relative expression levels, which were quantified using ImageJ software. Data represent mean ± SEM of four experiments. **P < 0.01; ***P < 0.001.

Next, we examined whether the dissimilar effect between CiMDA5 and CiRIG-I on IFN inducing was attributed to CiIRF3 and CiIRF7. To this end, we investigated the influence of CiMDA5 and CiRIG-I on CiIRF3 and CiIRF7 by luciferase activity assay, semiquantitative RT-PCR, and Western blotting. Before luciferase activity assay, the 5′-flanking sequences of CiIRF3 gene and CiIRF7 gene were proved to promote the expression of EGFP (Figure S3 in Supplementary Material). Following luciferase activity assay performed in FHM cells, we demonstrated that CiMDA5 inhibited the promoter activity of CiIRF3, but had no effect on that of CiIRF7 and that CiRIG-I enhanced the promoter activity of CiIRF3, but inhibited that of CiIRF7 (Figures 3A,B). Next, we investigated whether CiMDA5 and CiRIG-I affected the mRNA and protein expression levels of CiIRF3 and CiIRF7. Thus, total RNA samples from MDA5+ cells, RIG-I+ cells, and EGFP+ cells were isolated for the subsequent semiquantitative RT-PCR analysis. The result revealed that both CiMDA5 and CiRIG-I were able to positively regulate the mRNA expression of CiIRF3, but had no obvious effect on that of CiIRF7 (Figure 1A).

On the other hand, CiIRF7-His fusion protein was well expressed and purified, and then the purified protein was successfully identified by Western blotting (Figure S4A in Supplementary Material). With approximately one and half months of immunoreaction, the rabbit antiserum was collected. Before the subsequent Western blotting analysis, the rabbit anti-CiIRF7 antiserum was detected for its specificity according to the methods mentioned in the previous report (37), which showed that the anti-CiIRF7 antiserum rather than negative serum from the preimmunized rabbit was able to recognize a cellular protein with a molecular mass of ~49 kDa. When we preabsorbed anti-IRF7 antiserum with fusion protein IRF7-His as primary antibodies, the target protein band disappeared (Figure S4B in Supplementary Material). These data proved that the anti-CiIRF7 antiserum could be employed for further investigation. The following Western blotting analysis manifested that CiMDA5 and CiRIG-I affected the protein expression of neither CiIRF3 nor CiIRF7 (Figures 3C,D). Taken together, these data indicate that CiMDA5 and CiRIG-I influence CiIRF3 and CiIRF7 at the promoter activities and mRNA expression levels but not at protein expression levels.

### CiIRF3 and CiIRF7 Undergo Phosphorylation to Protect CIK Cells against GCRV Infection

**Figure 4:**
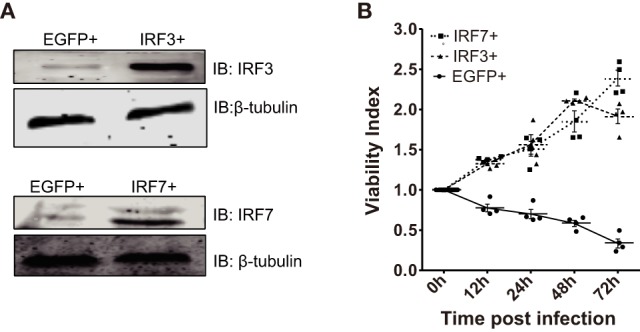
***Ctenopharyngodon idella* IFN regulatory factor 3 (CiIRF3) and CiIRF7 play positive roles in C. idella kidney cells under grass carp reovirus (GCRV) infection. (A)** IRF3+, IRF7+, and EGFP+ cells were seeded in 6-well plates for 24 h cultivation, and then the cell lysate was used for Western blotting analysis. **(B)** 3-(4,5-Dimethylthiazol-2-yl)-2,5-dimethyltetrazolium bromide (MTT) cell viability assay for GCRV-resistant testing in IRF3+, IRF7+, and EGFP+ cells. IRF3+, IRF7+, and EGFP+ cells were seeded in 96-well plates and then infected with GCRV. At each scheduled time, cell viability was analyzed by the MTT colorimetric method and expressed as viability index. Data represent mean ± SEM of four experiments.

**Figure 5:**
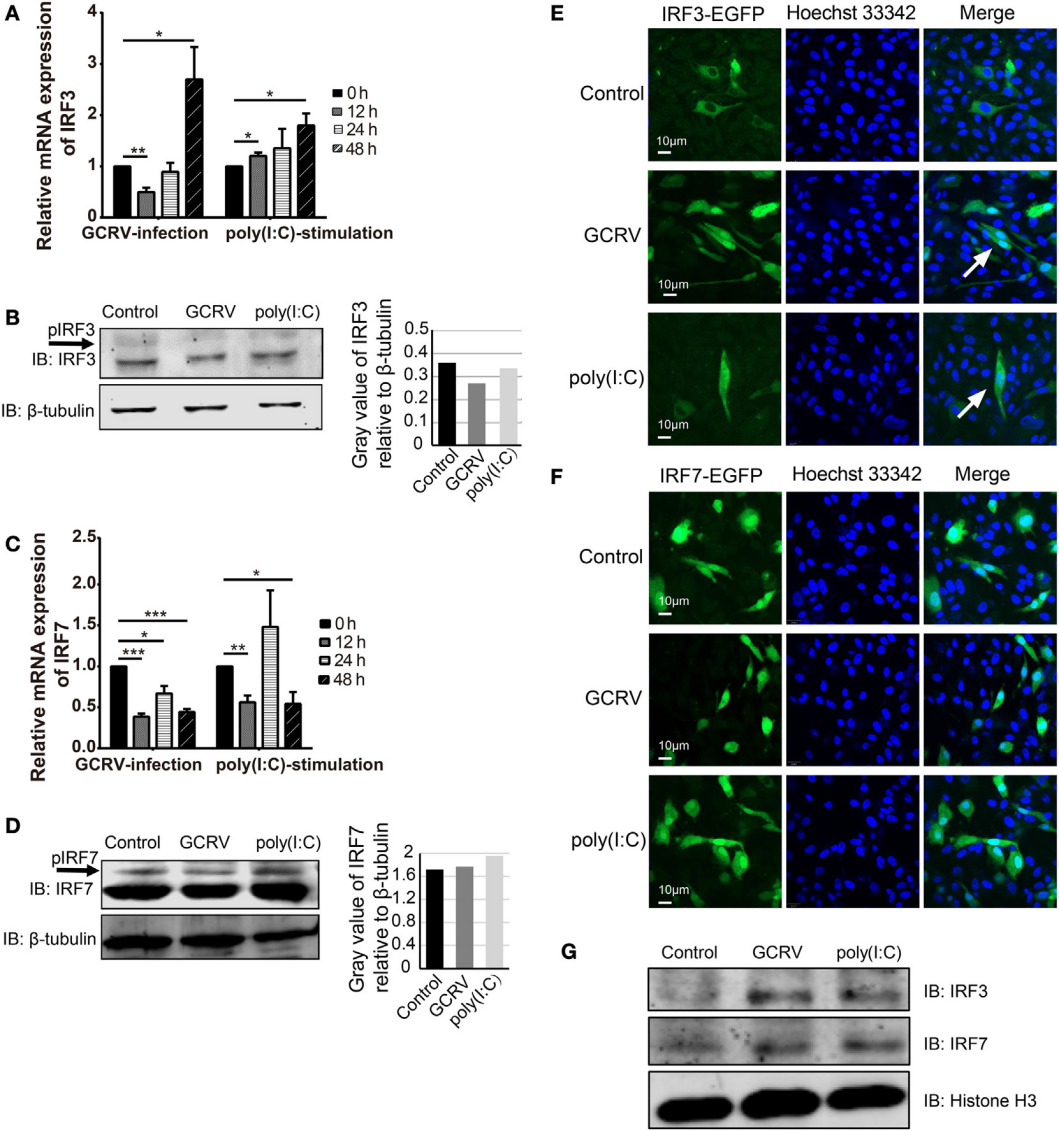
**Expression and subcellular localizations of grass crap IFN regulatory factor 3 (CiIRF3) and CiIRF7 are affected by grass carp reovirus (GCRV) infection or polyinosinic:polycytidylic acid [poly(I:C)] stimulation**. For mRNA expression, *Ctenopharyngodon idella* kidney (CIK) cells were seeded in 12-well plates and then infected with GCRV or stimulated with poly(I:C). At the scheduled time, total RNAs were isolated, and RT-qPCR assay for CiIRF3 **(A)** and CiIRF7 **(C)** was performed. The data are presented in relative expression units where *EF1*α was used to normalize all samples, and fold change was determined relative to cells before infection (0 h). Data represent mean ± SEM of four independent wells of cells. *P < 0.05; **P < 0.01; ***P < 0.001. For protein expression, CIK cells seeded in 6-well plates were lysed at 24 h post-GCRV infection or poly(I:C) stimulation, and then Western blotting analysis for CiIRF3 **(B)** and CiIRF7 **(D)** was carried out. The histograms exhibit the relative protein expression levels, which are quantified using ImageJ software. “p” in the front of IFN regulatory factor 3/7 indicates the phosphorylation. **(E,F)** Subcellular localization of CiIRF3 and CiIRF7. CIK cells that stably transfected with pIRF3-EGFP **(E)** or pIRF7-EGFP **(F)** were seeded on microscope coverslips in 12-well plates, followed by either phosphate-buffered saline (PBS), GCRV, or poly(I:C) treatment for 24 h. Then those cells were observed using a confocal fluorescence microscope (PerkinElmer). Arrows indicate nucleus translocation of CiIRF3. **(G)** CIK cells seeded in 6-well plates were either treated with PBS, GCRV, or poly(I:C). Twenty-four hours later, cells were harvested, and the nucleoprotein was prepared for the subsequent Western blotting analysis.

In consideration of the void effect of CiMDA5 and CiRIG-I on the protein expressions of CiIRF3 and CiIRF7, we wondered that whether CiIRF3 or CiIRF7 played a role in GCRV infection. Thus, either pIRF3 or pIRF7 was stably transfected into CIK cells (Figure 4A), and the viability of the stably transfected CiIRF3 (IRF3+) and CiIRF7 (IRF7+) cells upon GCRV infection was determined using an *ex vivo* cell viability assay. Cell proliferation in IRF3+ and IRF7+ cells, but cell death in EGFP+ cells were observed (Figure 4B), which attested the positive roles of CiIRF3 and CiIRF7 in CIK cells against GCRV infection. Next, we sought to investigate whether the expression levels and subcellular localization of CiIRF3 and CiIRF7 were affected by GCRV infection or poly(I:C) stimulation. At first, mRNA expression levels of CiIRF3 and CiIRF7 were detected by RT-qPCR. The mRNA level of CiIRF3 was provisionally inhibited at 12 h and then induced post-GCRV infection, while it was induced post-poly(I:C) stimulation in a time-dependent manner (Figure 5A). In contrast, the mRNA level of CiIRF7 was depressed under GCRV infection or poly(I:C) stimulation (Figure 5C). Second, in accordance with the mRNA expression patterns of CiIRF3 and CiIRF7, we performed the Western blotting at 24 h poststimulation. The results revealed that CiIRF3 and CiIRF7 were not visibly affected by GCRV infection or poly(I:C) stimulation, though the data analyzed by ImageJ software showed that CiIRF3 was inhibited and CiIRF7 was slightly increased (Figures 5B,D). Third, confocal fluorescence microscopy analysis was employed to investigate the subcellular localization of CiIRF3 and CiIRF7, which showed that CiIRF3 translocated from cytoplasm into nucleus (Figure 5E), while CiIRF7 persistently existed in the whole cell (Figure 5F). To verify the result of confocal fluorescence microscopy, we investigated the levels of CiIRF3 and CiIRF7 in the nucleus. The result showed that little CiIRF3 and much more CiIRF7 existed in the nucleus without immunostimulations, but they were aggregated into the nucleus under GCRV infection or poly(I:C) stimulation (Figure 5G). These results suggested that CiIRF3 and CiIRF7 were implicated into the immune response provoked by GCRV or poly(I:C) in CIK cells.

Accidently, we noticed that there were blurry blotting bands above the corresponding objective bands of CiIRF3 and CiIRF7, which might be the phosphorylation forms of CiIRF3 or CiIRF7. Therefore, a verification test was carried out. Briefly, the whole-cell lysis was incubated with or without 10 U of CIP before incubating with phosphoserine antibody, and the precipitates were detected by Western blotting. As anticipated, bands in the untreated group were abundant, while there was no band in the CIP treated group (Figure S4C in Supplementary Material). Subsequently, when we treated the whole-cell lysis with CIP, the larger bands above CiIRF7 disappeared (Figure S4D in Supplementary Material). Based on these confirmations, the Western blotting analysis revealed that the phosphorylated CiIRF3 and CiIRF7 were induced by GCRV infection or poly(I:C) stimulation. Taken together, these data show that CiIRF3 and CiIRF7 play positive roles through the phosphorylation and subsequent nuclear translocation (CiIRF3) in CIK cells infected with GCRV or stimulated with poly(I:C).

### CiMDA5 and CiRIG-I Differentially Regulate the Phosphorylation Status of CiIRF3 and CiIRF7 Post-GCRV Infection

**Figure 6:**
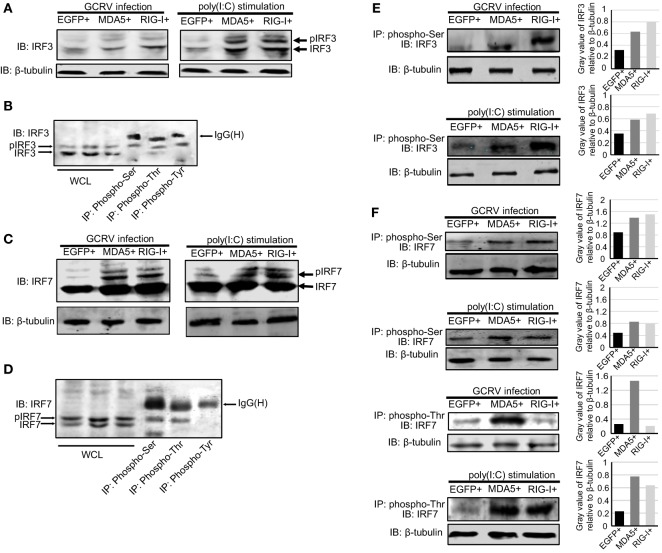
***Ctenopharyngodon idella* melanoma differentiation-associated gene 5 (CiMDA5) and *C. idella* retinoic acid-inducible gene I (CiRIG-I) affect the phosphorylation levels of grass carp IFN regulatory factor 3 (CiIRF3) and CiIRF7 under grass carp reovirus (GCRV) infection or polyinosinic:polycytidylic acid [poly(I:C)] stimulation. (A,C)** MDA5+, retinoic acid-inducible gene I (RIG-I)+, and EGFP+ cells were seeded into 6-well plates, and then either treated with phosphate-buffered saline, infected with GCRV, or stimulated with poly(I:C) for 24 h. Whole-cell lysates (WCL) were prepared for Western blot analysis. “p” in the front of IRF3\7 indicates the phosphorylation. **(B,D)** *C. idella* kidney cells were seeded in 10-cm plates, and then infected with GCRV for 24 h. WCL were subjected to immunoprecipitation (IP) with 1 μg of corresponding antiphosphorylation antibodies, followed by Western blotting analysis. **(E,F)** MDA5+, RIG-I+, and EGFP+ cells were seeded into 10-cm plates, and then either infected with GCRV or stimulated with poly(I:C). Twenty-four hours later, IP assays were performed with antiphosphoserine antibody or antiphosphothreonine antibody. The histograms exhibit the relative expression levels, which are quantified using ImageJ software. All the immunoblots were performed using anti-IRF3 or anti-IRF7 anti-rabbit polyclonal antibody.

To further explore whether CiMDA5 and CiRIG-I are correlative with CiIRF3 and CiIRF7 and the mechanism by which CiMDA5 and CiRIG-I influence CiIRF3 and CiIRF7, MDA5+, RIG-I+, and EGFP+ cells were either infected with GCRV or stimulated with poly(I:C). Twenty-four hours later, we found that the phosphorylation and protein expression levels of CiIRF3 and CiIRF7 were higher in MDA5+ and RIG-I+ cells than those in EGFP+ cells (Figures 6A,C). Subsequent IP assay showed that the serine, threonine, and tyrosine phosphorylation events occurred on CiIRF3, while serine and threonine phosphorylation events happened on CiIRF7 (Figures 6B,D). To verify the auxo-action of CiMDA5 and CiRIG-I on the phosphorylated activation of CiIRF3 and CiIRF7, we performed IP assay using phosphoserine and phosphothreonine antibodies. As expected, under GCRV infection or poly(I:C) stimulation, the phosphorylation levels of CiIRF3 and CiIRF7 in MDA5+ and RIG-I+ cells were higher than those in EGFP+ cells (Figures 6E,F). Moreover, CiRIG-I was able to facilitate a stronger phosphorylation of CiIRF3 than CiMDA5, while on the contrary, CiMDA5 facilitated a stronger phosphorylation of CiIRF7 than CiRIG-I. Taken together, these data strongly suggest that CiMDA5 and CiRIG-I facilitate not only the protein expressions but also the phosphorylated levels of CiIRF3 and CiIRF7 under GCRV infection or poly(I:C) stimulation.

### CiMDA5 and CiRIG-I Distinguishingly Modulate the Dimerization of CiIRF3 and CiIRF7, Leading to the Differential Production of IFNs

**Figure 7:**
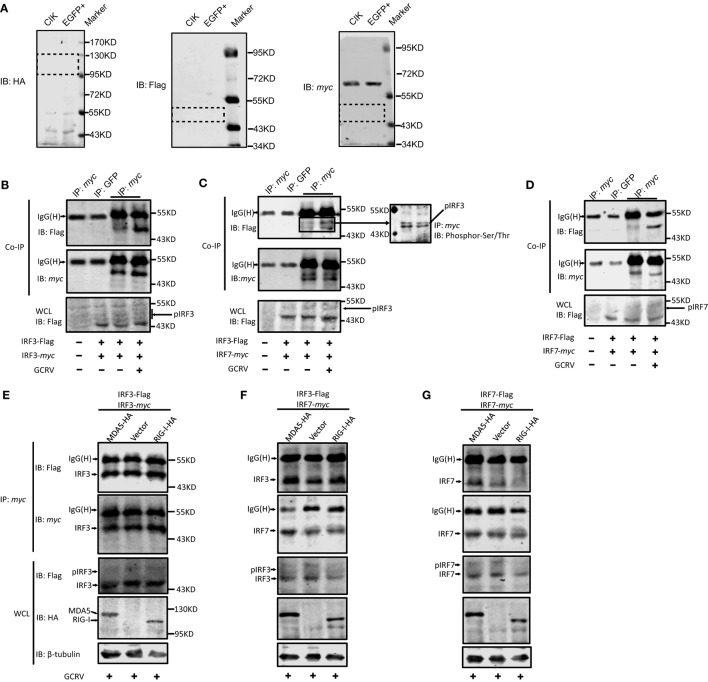
**The same and differential effects of *Ctenopharyngodon idella* melanoma differentiation-associated gene 5 (CiMDA5) and ***C. idella*** retinoic acid-inducible gene I (CiRIG-I) on the dimerization of grass carp IFN regulatory factor 3 (CiIRF3) and CiIRF7**. **(A)** Applicability test for anti-HA, -Flag, -*myc* antibodies. *C. idella* kidney (CIK) and EGFP+ cells seeded in 6-well plates were harvested for Western blot assay. Anti-HA, -Flag, -*myc* antibodies were used. Dotted boxes show the areas of corresponding target protein bands. **(B–D)** Grass carp reovirus (GCRV) infection facilitates the homo- and heterodimerization of IRF3 and IRF7. CIK cells seeded in 10-cm dishes were co-transfected with 4 μg pIRF3-Flag and 4μg pIRF3-*myc* **(B)**, pIRF3-Flag and pIRF7-*myc* **(C)**, and pIRF7-Flag and pIRF7-*myc* **(D)**. Twenty-four hours later, the transfected cells were infected with GCRV. At 24 h postinfection, cells were harvested for Co-IP assay with anti-*myc* antibody and subsequently immunoblotted with the indicated antibodies. **(E–G)** Effect of CiMDA5 and CiRIG-I on the dimerization of CiIRF3 and CiIRF7. CIK cells seeded in 10-cm dishes were co-transfected with 2.6 μg of pMDA5-HA (or pRIG-I-HA, or pdCMV), 2.6 μg of pIRF3-Flag and 2.6 μg of pIRF3-*myc* **(E)**, pIRF3-Flag and pIRF7-*myc* **(F)**, pIRF7-Flag and pIRF7-*myc* **(G)**. The subsequent experiments were carried out as described above.

As described above, CiMDA5 and CiRIG-I facilitated the phosphorylation levels of CiIRF3 and CiIRF7 at different degrees. Notably, we noticed that CiMDA5 increased but CiRIG-I reduced the threonine phosphorylation level of CiIRF7 upon GCRV infection, and CiMDA5 more potently enhanced it under poly(I:C) stimulation. Given that phosphorylation is a crucial step for the dimerization of IRF3 or IRF7 (10), CiMDA5 and CiRIG-I may differentially affect the dimerization of CiIRF3 and CiIRF7. To address this, we investigated the dimerization of CiIRF3 and CiIRF7 in either CiMDA5 or CiRIG-I overexpressed cells by Co-IP using tag antibodies whose applicability in CIK cells was verified. As shown in Figure 7A, in the respective target regions, proteins from CIK and EGFP+ cells could not be recognized by either anti-HA, anti-Flag, or anti-*myc* antibody, indicating these tag antibodies can be employed for subsequent experiments. Two sets of IRF3/7 constructs carrying the Flag or *myc* tag were co-transfected into CIK cells. In CiIRF3-Flag and CiIRF3-*myc*, CiIRF3-Flag and CiIRF7-*myc*, CiIRF7-Flag and CiIRF7-*myc* overexpressing cells, anti-*myc* antibody immunoprecipitated protein complex was immunoblotted by anti-Flag Ab. The anti-*myc* antibody immunoprecipitated protein complex from non-transfected cells and anti-GFP antibody immunoprecipitated protein complex from transfected cells were not able to be recognized by anti-Flag antibody (Figures 7B–D). The results indicated that CiIRF3 and CiIRF7 were able to shape homodimers and heterodimers. Notably, in anti-*myc* antibody immunoprecipitated protein complex from CiIRF3-Flag and CiIRF7-*myc* overexpressing cells, two protein bands were immunoblotted by anti-*myc* and anti-Flag antibodies. As shown in Figure 7C, the anti-*myc* antibody immunoprecipitated protein complex was recognized by serine- and threonine phosphorylation antibodies, indicating that both bands were phosphorylated proteins. Furthermore, it was observed that CiIRF3-Flag was weakly combined with CiIRF3-*myc* in the steady state, whereas they strongly combined under GCRV infection in CIK cells (Figure 7B). The same was true for the combinations between CiIRF3-Flag and CiIRF7-*myc* and between CiIRF7-Flag and CiIRF7-*myc* (Figures 7C,D), thus demonstrating that GCRV infection strengthened both homo- and heterodimerization of CiIRF3 and CiIRF7.

Considering that dimerization of CiIRF3 and CiIRF7 was strengthened by GCRV infection, the dimerization in grass carp plays a critical role in IFN induction, same as that in mammalian. Hereupon, we wondered whether CiMDA5 and CiRIG-I differentially affected the dimerization of CiIRF3 and CiIRF7. Therefore, constructs were transiently co-transfected into CIK cells, IP of *myc*-tagged protein was performed, followed by immunoblotting analysis of the immunoprecipitate with anti-Flag antibody and anti-*myc* antibody, respectively. As shown in Figure 7E, the amount of Flag-tagged CiIRF3 in anti-*myc* antibody immunoprecipitate from CiMDA5- or CiRIG-I overexpressed cells was equal to that from empty vector transfected cells, revealing that CiMDA5 and CiRIG-I were not able to affect the homodimerization of CiIRF3. Similar experiments illuminated that CiMDA5 and CiRIG-I facilitated the heterodimerization of CiIRF3 and CiIRF7 (Figure 7F). Dissimilarly, CiMDA5 facilitated but CiRIG-I impeded the homodimerization of CiIRF7 (Figure 7G).

**Figure 8:**
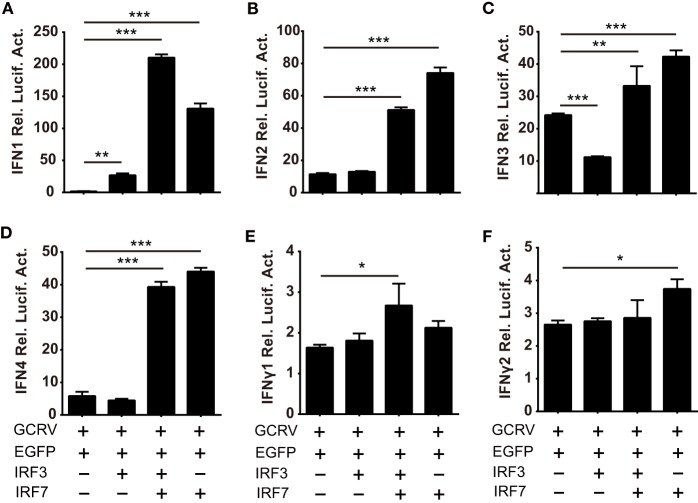
**Differential responses of IFN promoters to ***Ctenopharyngodon idella*** IFN regulatory factor 3/7 (CiIRF3/7) individually and collectively**. *C. idella* kidney cells seeded in 24-well plates were co-transfected with 380 ng pIFNpro-Luc [either pIFN1pro-Luc **(A)**, pIFN2pro-Luc **(B)**, pIFN3pro-Luc **(C)**, pIFN4pro-Luc **(D)**, pIFNγ1pro-Luc **(E)**, or pIFNγ2pro-Luc **(F)**], and 380 ng indicated plasmids [pIRF3 the second bar of each figure, pIRF3:pIRF7 = 1:4 the third bar of each figure, pIRF7 the fourth bar of each figure] and 38 ng pRL-TK. Twenty-four hours later, the cells were infected with GCRV. The luciferase activities were detected at 6 h p.i. Error bars represent SEMs obtained by testing each sample in quadruplicate. *P < 0.05; **P < 0.01; ***P < 0.001.

Based on the abovementioned data, we ought to verify the assumption that the homodimer and heterodimer of CiIRF3 and CiIRF7 differentially induce CiIFNs production. As a consequent, luciferase assays were employed to test the expression regulation of CiIFNs by CiIRF3 and CiIRF7. To examine the synergistic effect between CiIRF3 and CiIRF7, pIRF3 and pIRF7 were co-transfected into CIK cells at a ratio of1:4, which had been proved to be the perfect ratio in zebrafish (40). As shown in Figure 8, single CiIRF3 overexpression significantly activated the promoter of CiIFN1, but suppressed the promoter of CiIFN3, and failed to activate the promoters of CiIFN2 and CiIFN4. Single CiIRF7 overexpression or both CiIRF3 and CiIRF7 overexpression widely activated the promoters of IFN-I, and the synergy between CiIRF3 and CiIRF7 was embodied in the activation of CiIFN1 promoter. Consistent with previous theory that IRF3 and IRF7 induced the production of IFN-I but not IFN-II, overexpressed CiIRF3/7 alone or collectively were not able to activate the promoters of IFNγ1 and IFNγ2 (Figures 8E,F). These data demonstrate that the different effect between CiMDA5 and CiRIG-I on IFN-I inducing is involved in the phosphorylation and dimerization of CiIRF3 and CiIRF7.

### CiMDA5 and CiRIG-I Differentially Affect the mRNA Expressions of CiIL-4 and CiIL-12p40

**Figure 9:**
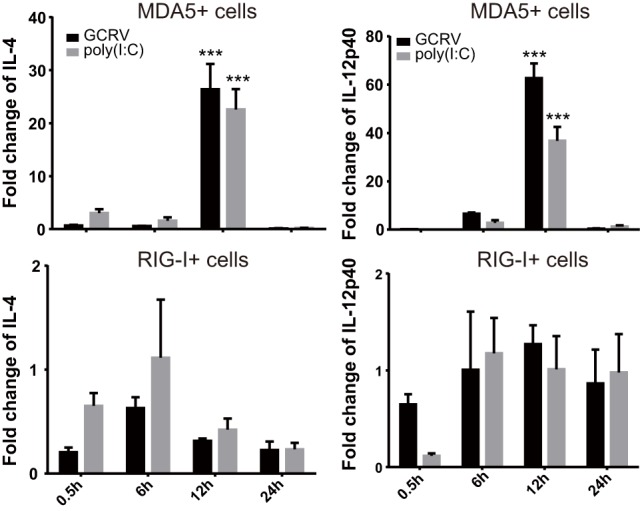
***Ctenopharyngodon idella* melanoma differentiation-associated gene 5 (CiMDA5) and ***C. idella*** retinoic acid-inducible gene I (CiRIG-I) differentially affect the mRNA expression levels of CiIL-4 and CiIL-12p40 genes**. *C. idella* kidney (CIK), MDA5+, and RIG-I+ cells were seeded in 12-well plates, and then infected with GCRV or stimulated with polyinosinic:polycytidylic acid. At the scheduled time, total RNA samples were isolated for RT-qPCR analysis. The relative expression levels of these genes were normalized by *EF1*α. Fold change of gene expression level was determined relative to corresponding treated CIK cells at the same time point. Data represent mean ± SEM of four independent wells of cells. ***P < 0.001.

We further attempted to inquire the possible mechanism by which CiMDA5 and CiRIG-I differentially induced the expression of CiIFN-II. Given that certain cytokines such as IL-4, IL-10, IL-12, and IL-18 can regulate IFN-II production (41), we wondered whether CiMDA5 and CiRIG-I differentially affected the expression of such cytokines. Hence, we obtained the sequences of CiIL-4 gene (GenBank accession no. KP896505) and CiIL-10 gene (GenBank accession no. HQ388294), but did not find the sequence of CiIL-18 gene in GenBank or the genomic database of grass carp (32). Subsequently, we designed primers of CiIL-4 and CiIL-10 and used available primers of CiIL-12p40 (38) for the following RT-qPCR assay (Table 1). As shown in Figure 9, compared to the expression levels in CIK cells, CiIL-4 and CiIL-12p40 were significantly highly expressed in MDA5+ cells at 12 h post-GCRV infection or poly(I:C) stimulation. However, the expression of CiIL-4 was repressed in RIG-I+ cells, the expression of CiIL-12p40 in RIG-I+ cells was same as that in CIK cells. Unexpectedly, CiIL-10 was not detected in all samples, suggesting that CiIL-10 was hardly expressed in CIK cells. These data reveal that CiMDA5 increases the expression of CiIL-4 and CiIL-12p40, while CiRIG-I represses that of CiIL-4, which may provide clues for the further studies on the correlation between RLRs and IFN-II.

## Discussion

As critical intracellular sensors, the recognition mechanisms of MDA5 and RIG-I were broadly investigated in the past few years. To date, a widespread theory considers that MDA5 and RIG-I play non-redundant roles in pathogen recognition: MDA5 recognizes positive single strand RNA (ssRNA) or long double strand RNA (dsRNA) virus, while RIG-I recognizes negative ssRNA or short dsRNA virus (2). This conclusion signifies that MDA5 and RIG-I jointly recognize those viruses whose genome is composed of different-sized segmented dsRNA, such as reovirus. In this case, MDA5 and RIG-I are seemingly redundant sensors in antiviral immunity upon such virus invasion, and thus their shared and/or unique functions in the downstream signal pathway should be elucidated. Moreover, previous studies had stated or identified that distinct pathogens or virus MOI affected IFN-I subtype induction (42, 43), but the mechanisms are unclear. Based on the abovementioned observations, it is presumed that MDA5 and RIG-I induce differential IFN subtypes in antiviral immunity. Fish can be served as an appropriate research object because RIG-I homologs are absent in some fish genomes. To this end, a dsRNA reovirus named GCRV, which was previously proved to induce the expression of CiMDA5 and CiRIG-I *in vivo* and *in vitro*, served as stimulus of CIK cells in the present study (28, 29), aiming at revealing the similar and different roles between CiMDA5 and CiRIG-I in the induction of IFN system.

Though our previous studies reported that either CiMDA5 or CiRIG-I overexpression plays a positive role in antiviral response in CIK cells (27, 30), the distinction in IFN induction between them has yet to be clarified. CiIFN genes are recently sorted out by bioinformatics analysis (31), the expression patterns of CiIFNs were first investigated in the present study. The expression pattern of IFN-I in CIK cells is different from that in salmon cells, in which group I IFN-I (IFN-a, IFN-d, and IFN-e) but not group II IFN-I (IFN-b, IFN-c, and IFN-f) are ubiquitously produced and are inducible (IFN-a, IFN-d, IFN-e, and IFN-f) in response to viral infection (12). CiIFN3 is downregulated until 12 h p.i., indicating that CiIFN3 is inhibited at the early stage of GCRV infection. Besides, CiIFN4 is different from IFN-d (CiIFN4 homolog) in Atlantic salmon, which was identified to have no response to poly(I:C) (44). Likewise, IFN4 in rhabdoviruses infected zebrafish expressed lowly and shows low biological activity (45). Though the specific biological activity of CiIFN4 has yet to be investigated, its expression pattern implies that CiIFN4 plays a positive role in antiviral immunity. These data reveal that IFN1 and IFN3 are the major functional IFN-I, and the four IFN-I subtypes may play mutual synergistic and supplementary roles in CIK cells as previously reviewed (16). With regard to IFN-II, the moderate expression patterns may indicate their indispensable roles. Under immunostimulation, CiIFNγ1 could not be detected until 24 h p.i., and CiIFNγ2 is inhibited until 24 h p.i., implying that CiIFNγ1 and CiIFNγ2 play critical roles in antiviral immunity and are restrained at early stage in CIK cells. On the other hand, either CiMDA5 or CiRIG-I overexpression does not lead to the upregulation of IFNs at steady state in CIK cells, but significantly induces the expression of IFN-I upon GCRV or poly(I:C) stimulation, which may be attributed to the autoinhibition and phosphorylation of CiMDA5 and CiRIG-I at the steady state (46). Previous study on IFN-I in Atlantic salmon showed that IFN-a (CiIFN1 homolog) is the main IFN subtype induced by the RIG-I/MDA5 pathway (44). However, we found that CiIFN3 is dramatically induced by overexpressed CiMDA5 and CiRIG-I, indicating that RIG-I and MDA5 mainly induce the expression of IFN3 in grass carp. Comparing the expression patterns of various IFNs in MDA5+ cells with those in RIG-I+ cells, we found that the relative expression levels of IFNs in MDA5+ cells are higher than those in RIG-I+ cells. More obviously, CiMDA5 but not CiRIG-I overexpression upregulated the expression of CiIFN1, implying that the downstream signal pathway of MDA5 is not identical with that of RIG-I in grass carp. Subsequently, these consequences are validated by a luciferase reporter assay. CiMDA5 and CiRIG-I overexpressions are not able to activate the promoters of CiIFN-I at the steady state, but they activate the promoters under GCRV infection and, more importantly, CiMDA5 is more potent to activate CiIFN-I promoters than CiRIG-I. Furthermore, the expression patterns of ISG also underscored this conclusion. CiMDA5 overexpression induced higher expression levels of CiGig1, CiIFI56, and CiMx2 than CiRIG-I overexpression under immunostimulations. Of note is that, CiMDA5 and CiRIG-I overexpression cannot upregulate the expression of CiISG15. Previous study identified that ISG15 was able to conjugate with RIG-I to negatively regulate RIG-I-mediated antiviral signaling (47). Thus, we deduce that ISG15 is mainly induced by other signaling pathways to regulate the RLR pathway in grass carp. Additionally, we also noticed that CiMDA5 and CiRIG-I induce the mRNA expression of IFNγ2. Considering that IL-4 and IL-10 negatively regulate while IL-12 and IL-18 positively regulate the production of IFNγ, we attempted to detect the expression of these ILs. Since IL-18 is not retrieved in grass carp genome and the expression of CiIL-10 is not detected, we showed the expression profiles of CiIL-4 and CiIL-12p40. Considering NF-κB which initiates the transcriptions of various cytokines including some ILs and is a critical downstream transcription factor for MAVS (48, 49), CiMDA5 and CiRIG-I may affect the expressions of these ILs to regulate the production of IFN-II through the NF-κB pathway.

One probable mechanism for the differential effects between CiMDA5 and CiRIG-I is that CiRIG-I inhibits the expression of CiMDA5 (Figure 1A). The inhibition mechanism can be speculated from the distinct structures between MDA5 and RIG-I. As is well known that RIG-I but not MDA5 in a strictly autoinhibited conformation in the ligand-free state, the superabundant MDA5 in cytosol can lead to overactive immune response or apoptosis to threaten the cell survival (50, 51). Therefore, to maintain the intracellular balance, MDA5 was downregulated by a present unknown mechanism when RIG-I overexpressed. However, the autoinhibited RIG-I posed no threat to the cell survival, thus it is needless to regulate the expression of RIG-I when MDA5 overexpressed. This observation may result from a host adaption mechanism defined as “PRR reprogramming” (52). This finding indicates that the expression levels of MDA5 and RIG-I were in dynamic equilibrium in cytosol, and sometimes they play reciprocal inhibited roles. On the other hand, as key transcription factors of IFN-I in the RLR signaling pathway, IRF3 and IRF7 may be responsible for the functional differentiation between CiMDA5 and CiRIG-I. In zebrafish, IRF3 overexpression effectively activates IFN1, while IRF7 overexpression activates IFN3 (9), which implies that fish IRF3 and IRF7 play distinct roles in IFN signaling. Thus, it is doubtful whether CiMDA5 and CiRIG-I differently affect the expression of CiIRF3 and CiIRF7. Though the promoter activity and mRNA level of CiIRF3 and the promoter activity of CiIRF7 were affected by CiRIG-I and/or CiMDA5, the protein levels of CiIRF3 and CiIRF7 were not affected. Two research findings can be employed to understand the discordance among the promoter activity, mRNA and protein levels. First, transiently transfection can result in temporal changes in the expression of many transcripts (53). CiMDA5 and CiRIG-I were transiently overexpressed in the luciferase activity assay, while they were stably overexpressed in the RT-qPCR and Western blotting tests. Thus, we deduce that this discordance is caused by the overexpressed modes of CiMDA5 and CiRIG-I. Second, the discordance between protein and mRNA expression is prevalent in human, especially for genes of regulation in terms of biological process, which refers to many posttranscriptional mechanisms (54, 55). Overall, we found that CiRIG-I and CiMDA5 overexpression were able to affect the expression of CiIRF3 and CiIRF7 in CIK cells without immunostimulations. It is well known that immune system should stay in homeostasis without xenogeneic invasion. To avoid provoking the IFN response in the steady state, excrescent IRF3 and IRF7 should be degraded by certain mechanisms such as RNA degradation and protein ubiquitylation (56–58). On the other hand, considering IRF7 is majorly induced by IFN-I (59), the non-effect of CiMDA5 and CiRIG-I on CiIRF7 might be caused by the non-effect of CiMDA5 and CiRIG-I on IFN-I without immunostimulation. Based on such findings, we wondered whether CiIRF3 and CiIRF7 play roles in antiviral response in CIK cells. The subsequent investigation demonstrates that CiIRF3 and CiIRF7 indeed play protective roles in GCRV infection and that the expression levels of CiIRF3 and CiIRF7, as well as the subcellular location of CiIRF3, were affected by GCRV infection or poly(I:C) stimulation.

Since CiIRF3 and CiIRF7 participated in the antiviral response, it was still a puzzle that how CiMDA5 and CiRIG-I differently affected the downstream signals. Excitedly, we noticed that there are some faint slow-migrating bands above the target bands. Considering that IRF3 and IRF7 may be activated by phosphorylation, those larger bands might be the phosphorylation forms of CiIRF3 or CiIRF7. Subsequently, this hypothesis is verified by the IP and Western blotting assays. Therefore, we demonstrated that GCRV infection or poly(I:C) stimulation facilitates the phosphorylation levels of CiIRF3 and CiIRF7, which leads to CiIRF3 and CiIRF7 translocation from cytoplasm to the nucleus. More importantly, the expression and phosphorylation levels of CiIRF3 and CiIRF7 are higher in MDA5+ and RIG-I+ cells than those in EGFP+ cells under GCRV infection or poly(I:C) stimulation (Figure 6), indicating that the immunostimulation but not the expression of immune-related genes is the key to initiate immune responses. Subsequently, the effects of CiMDA5 and CiRIG-I on the phosphorylation levels of CiIRF3 and CiIRF7 are explored. The result shows that CiMDA5 and CiRIG-I significantly facilitate the serine phosphorylation levels of CiIRF3 and CiIRF7 under GCRV infection or poly(I:C) stimulation. Differently, since RIG-I is able to interact with STING to specify IRF3 phosphorylation (60, 61), CiRIG-I induces a higher phosphorylation level of CiIRF3 than CiMDA5. Interestingly, the threonine phosphorylation of CiIRF7 was facilitated by CiMDA5 but inhibited by CiRIG-I under GCRV infection, and the threonine phosphorylation level of CiIRF7 in CiRIG-I overexpressed cells was slightly lower than that in CiMDA5 overexpressed cells under poly(I:C) stimulation.

As it is well-known that phosphorylated IRF3 and IRF7 translocate into the nucleus and then form dimers to bind various IFN promoters, Teleost IRF3 prefers to bind the promoter and induce the production of IFN1, while IRF7 has a preference to induce IFN3 (9, 40). Therefore, the different dimerization forms of IRF3 and IRF7 induce different IFNs. However, which factor determines the dimerization form namely homo- or heterodimer is unknown. We posit that this factor is phosphorylation form. In light of the different effects between CiMDA5 and CiRIG-I on the production of CiIFNs and the phosphorylation of CiIRF3 and CiIRF7, we deduced that CiMDA5 and CiRIG-I differentially affect the dimerization of CiIRF3 and CiIRF7. Subsequent Co-IP assays verify this assumption. CiMDA5 and CiRIG-I facilitate the phosphorylation of CiIRF3 but have no obvious effect on the homodimerization of CiIRF3, while enhance the heterodimerization of CiIRF3 and CiIRF7, indicating that most CiIRF3 is employed to shape heterodimers with CiIRF7. More importantly, CiRIG-I increases the serine phosphorylation but decreases the threonine phosphorylation levels of CiIRF7, leading to the upregulation of the heterodimerization level of CiIRF3 and CiIRF7 but the downregulation of the homodimerization level of CiIRF7 under GCRV infection. It was previously stated that RIG-I but not MDA5 is autoinhibited, which gives MDA5 a greater propensity to form filaments along dsRNA (62). This difference may result in the more potent role of MDA5 than that of RIG-I in the immune response. Herein, the present results show the differential effect between CiMDA5 and CiRIG-I on the regulation of phosphorylation and dimerization of CiIRF3 and CiIRF7, revealing that CiMDA5 is more potent to modulate IFN-I production than CiRIG-I and, to a certain extent, CiMDA5 and CiRIG-I play redundant and complementary roles in the immune response against the invasion of some viruses which can be recognized by MDA5 and RIG-I. On the other hand, these results suggest that threonine phosphorylation of CiIRF7 is required to shape CiIRF7 homodimers, while serine phosphorylation of CiIRF7 is required to shape CiIRF7 heterodimers with CiIRF3. According to this inference, CiIRF7 may dominate the heterodimerization between CiIRF3 and CiIRF7. Notably, subsequent dual luciferase reporter assay reveals that the heterodimer of CiIRF3 and CiIRF7 strongly activate the promoters of IFN-I genes, emphasizing the critical role of the heterodimerization in IFN-I induction. In the process of heterodimerization, another unknown protein modification is required. The direct evidence for this statement is that there are two different sizes of phosphorylated CiIRF3-Flag and phosphorylated CiIRF7-*myc* in the immunoprecipitate from CiIRF3-Flag and CiIRF7-*myc* overexpressed cells (Figure 7C). Coincidentally, similar result is found in zebrafish (40). Given that most phosphorylation residues of human and mouse IRF3/7 are not conservative in CiIRF3/7 (Figure S5 in Supplementary Material), to clarify the phosphorylation residues of CiIRF3/7 may be of great interest in the future, which may contribute to understanding the fine regulatory mechanisms of the dimerization. Similar to previous reports in zebrafish (9, 40), IRF3 prefers to activate IFN1, while IRF7 widely activates IFN-I genes in grass carp. Previously, Honda and Taniguchi deduced that the homodimer of IRF7 or the heterodimer of IRF7 and IRF3, rather than the homodimer of IRF3, might be more important for the cytosolic pathway of IFN-I gene induction by viruses (10). Consistently, we found that IRF7 is more efficient in IFN-I promoter activation than IRF3 in grass carp. In light of the abovementioned results, we approved the viewpoint that IRF7 plays a more important role in the pathway of production of IFN-I than IRF3 under virus infection (10, 63). Thus, the IFN-I response to GCRV infection in CIK cells can be conceived as that: IRF3 is activated for the shaping of homodimer to induce IFN1 production, and then IFN1 induces the expression and activation of IRF7 which then forms homodimers and heterodimers with IRF3 to induce stronger expression of IFN1, IFN2, IFN3, and IFN4.

**Figure 10:**
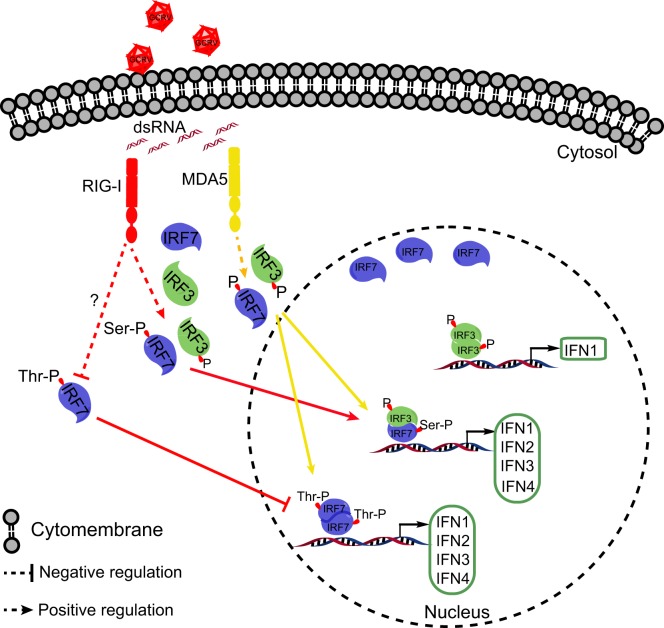
**Schematic representation of the difference between *Ctenopharyngodon idella* melanoma differentiation-associated gene 5 (CiMDA5) and ***C. idella*** retinoic acid-inducible gene I (CiRIG-I) in inducing grass carp IFNs (CiIFN) genes**. The red arrows indicate the RIG-I pathway, while yellow arrows indicate the MDA5 pathway. Under grass carp reovirus (GCRV) infection, CiMDA5 and CiRIG-I facilitate the holistic phosphorylation levels of grass carp IFN regulatory factor 3 (CiIRF3) and CiIRF7. Both CiMDA5 and CiRIG-I enhance heterodimerization of CiIRF3 and CiIRF7. Differently, CiMDA5 facilitates but CiRIG-I weakens the threonine phosphorylation level of CiIRF7, which results in the status that CiMDA5 facilitates but CiRIG-I weakens the homodimerization of CiIRF7. This mechanism explains that CiMDA5 induces a stronger CiIFN-I response than CiRIG-I under GCRV infection in *Ctenopharyngodon idella* kidney cells.

Collectively, the present study revealed that CiMDA5 induces a more extensive IFN response than CiRIG-I under GCRV infection. As shown in Figure 10, the differential effect is ascribed to the following mechanisms. CiMDA5 facilitates the total phosphorylation levels of CiIRF3 and CiIRF7, while CiRIG-I facilitates the total phosphorylation level of CiIRF3 and serine phosphorylation level of CiIRF7 but decreases the threonine phosphorylation level of CiIRF7. This difference gives rise to the observation that CiMDA5 enhances the heterodimerization of CiIRF3 and CiIRF7, and the homodimerization of CiIRF7; while CiRIG-I strengthens the heterodimerization of CiIRF3 and CiIRF7, but impairs the homodimerization of CiIRF7. Since CiIRF7 homodimer broadly induces the production of IFN-I, CiRIG-I induces a weaker IFN-I response than CiMDA5 upon GCRV infection. These findings imply that CiMDA5 is crucial for the cytosolic pathway in induction of IFN genes by GCRV, and that the contribution of CiRIG-I is minor. This may be another evidence to explain why MDA5 is able to completely substitute RIG-I in Chinese tree shrew (64). Under this situation, the matter that RIG-I cannot be identified in certain fishes can be well explained.

## Author Contributions

JS and QW conceived and designed the experiments. QW, CY, and YR performed the experiments and analyzed the data. ZL contributed to the bioinformatics analysis of correlative gene sequences. QW and JS wrote the manuscript. All authors reviewed the manuscript.

## Conflict of Interest Statement

The authors declare that the research was conducted in the absence of any commercial or financial relationships that could be construed as a potential conflict of interest.
