# Supplementary material for: MDA5 Induces a Stronger Interferon Response than RIG-I to GCRV Infection through a Mechanism Involving the Phosphorylation and Dimerization of IRF3 and IRF7 in CIK Cells
Source: Front Immunol. 2017 Feb 24;8:189. doi: 10.3389/fimmu.2017.00189 (PMC5323377; doi:10.3389/fimmu.2017.00189)
Supplement: Supplementary file 1 [file data_sheet_1.docx]

Supplemental Table I Primers for plasmids construction.

| Primer name | Primer sequence (5'–3') | Constructs |
| --- | --- | --- |
| IF116  IR117 | **CG***GAATTC*ATGACCCATCCAAAACCG  **CG***GGTACC*TCACTTGGTGTCACACAACTC | pIRF3 |
| IRR566 | **GG***GGTACC*TCACTTATCGTCGTCATCCTTGTAATCCTTGGTGTCACACAACTCCAT | pIRF3-Flag |
| IRR578 | **GG***GGTACC*TCACAGATCCTCTTCAGAGATGAGTTTCTGCTCCTTGGTGTCACACAACTCCAT | pIRF3-Myc |
| IF142  IR144b | **AGTC***GGTACC*ATGACCCATCCAAAACCG  **AGTC***GGGCCC*CACTTGGTGTCACACAACTC | pIRF3-EGFP |
| IF1796  IR1827 | **ACTG***CTCGAG*TGGTAAAGACCAAAGAGAGGGAG  **ACTG***AAGCTT*GTTGCAAACAGACTACACAACACA | pIRF3pro-EGFP and  pIRF3pro-Luc |
| IF118a  IR133 | **CG***GAATT*CAGACCTGAAATCATGGCAG  **ACCG***CTCGAG*CTTTAGTCCATTGAAGGCAG | pET-32a(+)-IRF7 |
| IF118a  IR119 | **CG***GAATT*CAGACCTGAAATCATGGCAG  **CG***GGTACC*CTTTAGTCCATTGAAGGCAG | pIRF7 |
| IRR567 | **GG***GGTACC*TTACAGATCCTCTTCAGAGATGAGTTTCTGCTCGTCCATTGAAGGCAGA | pIRF7-Myc |
| IRR579 | **GG***GGTACC*TTACTTATCGTCGTCATCCTTGTAATCGTCCATTGAAGGCAGACC | pIRF7-Flag |
| IF138  IR139b | **AGTC***GGTACC*AAGACCTGAAATCATGGCAG  **AGTC***GGATCC*CTTTTGTCCATTGAAGGCAG | pIRF7-EGFP |
| IF140  IR141 | **ACTG***CTCGAG*GTCTTCAGTGCCACATGATC  **ACTG***AAGCTT*TTCAGGTCTTGAAGTAGCTT | pIRF7pro-EGFP and  pIRF7pro-Luc |
| IFF568  IFR569 | **GA***agatct*TACCAAAACCAGAGCCAAGAG  **CCC***AAGCTT*AAGGAACGGAGAGCGTATGA | pIFN3pro-EGFP and pIFN3pro-Luc |
| IFF570  IFR571 | **GA***agatct*TGGGAAATGAACGCAAGGT  **CCC***AAGCTT*TCCACAGGTAGATGAGCCG | pIFN4pro-EGFP and pIFN4pro-Luc |

Note: Italic letters indicate the accessional restriction enzyme cutting sites, bold letters indicate the protective bases.


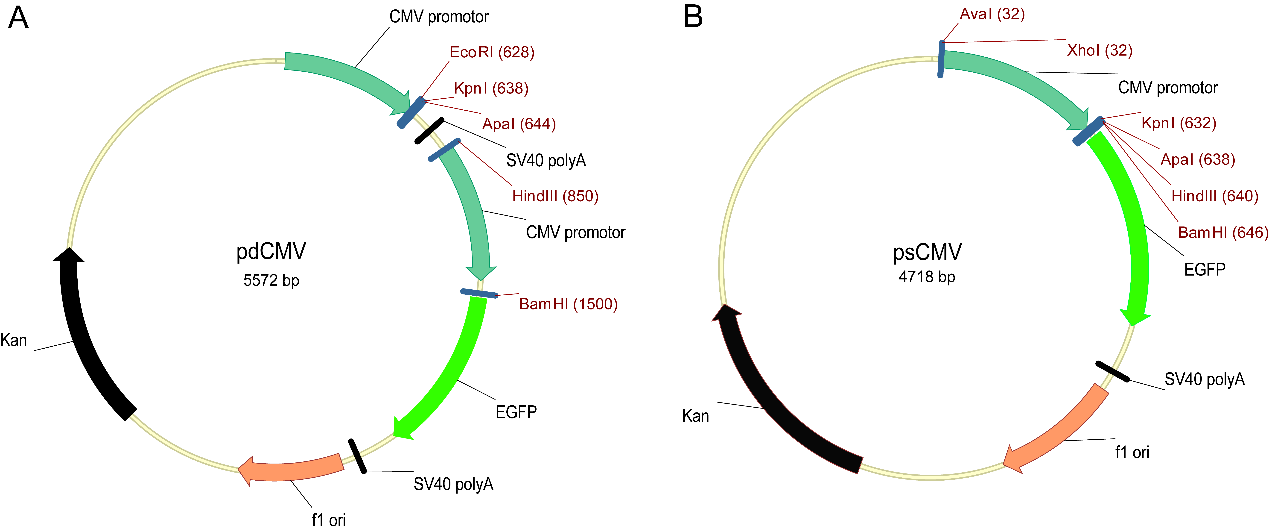


**Supplemental FIGURE 1 Illustration of plasmids**. **(A)** pdCMV, contains double CMV promoter which can independently promote the expression of inserted gene and EGFP; **(B)** psCMV, contains single CMV promoter which drive the expression of downstream EGFP fusion protein.


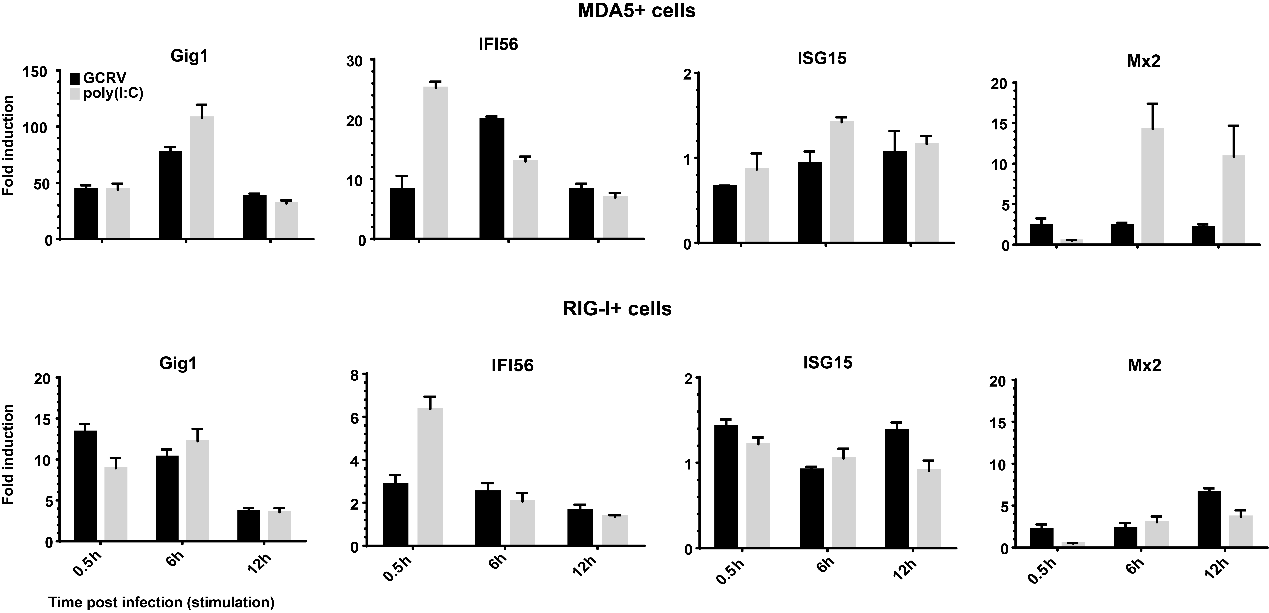


**Supplemental FIGURE 2 CiMDA5 and CiRIG-I differentially induce the production of ISGs.** CIK, MDA5+, and RIG-I+ cells were seeded in 12-well plates, and then were either PBS treated, GCRV infected, or poly(I:C) stimulated. Total RNA samples were isolated at the scheduled time post-challenge. The relative expression levels of these genes were normalized by *EF1α*. Fold induction of gene expression level in MDA5+ and RIG-I+ cells were determined relative to corresponding treated CIK cells at the same time point. Data represent mean ±SEM of four independent wells of cells.


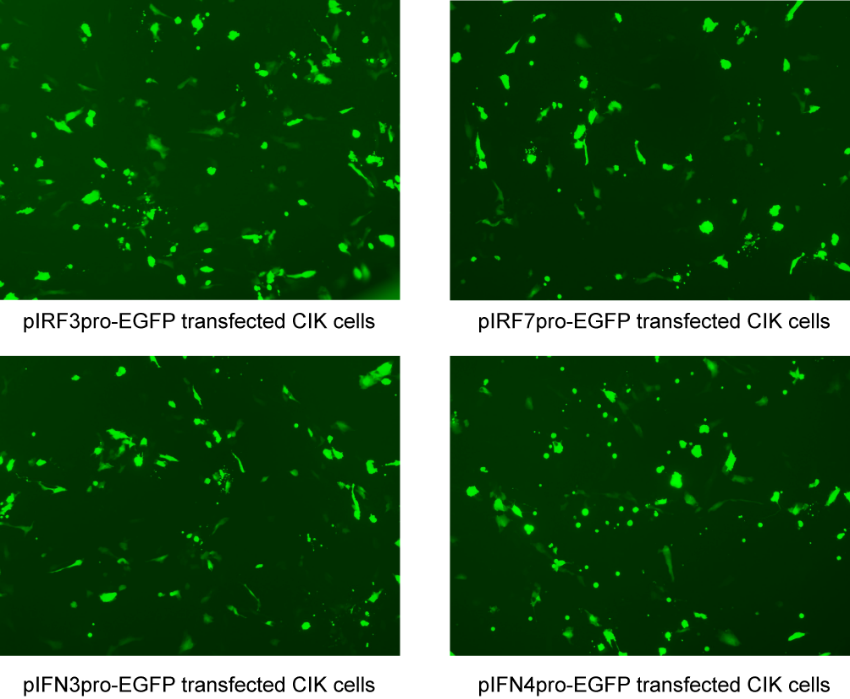


**Supplemental FIGURE 3 Observation results for verifying the promoter activity of the 5'-flanking region of CiIRF3, CiIRF7, CiIFN3, and CiIFN4 by fluorescent microscopy**. The CIK cells transfected with pIRF3pro-EGFP, pIRF7pro-EGFP, pIFN3pro-EGFP, pIFN4pro-EGFP were observed under fluorescent microscopy. These green fluorophores are the EGFPs. Magnification: 10 × 10.


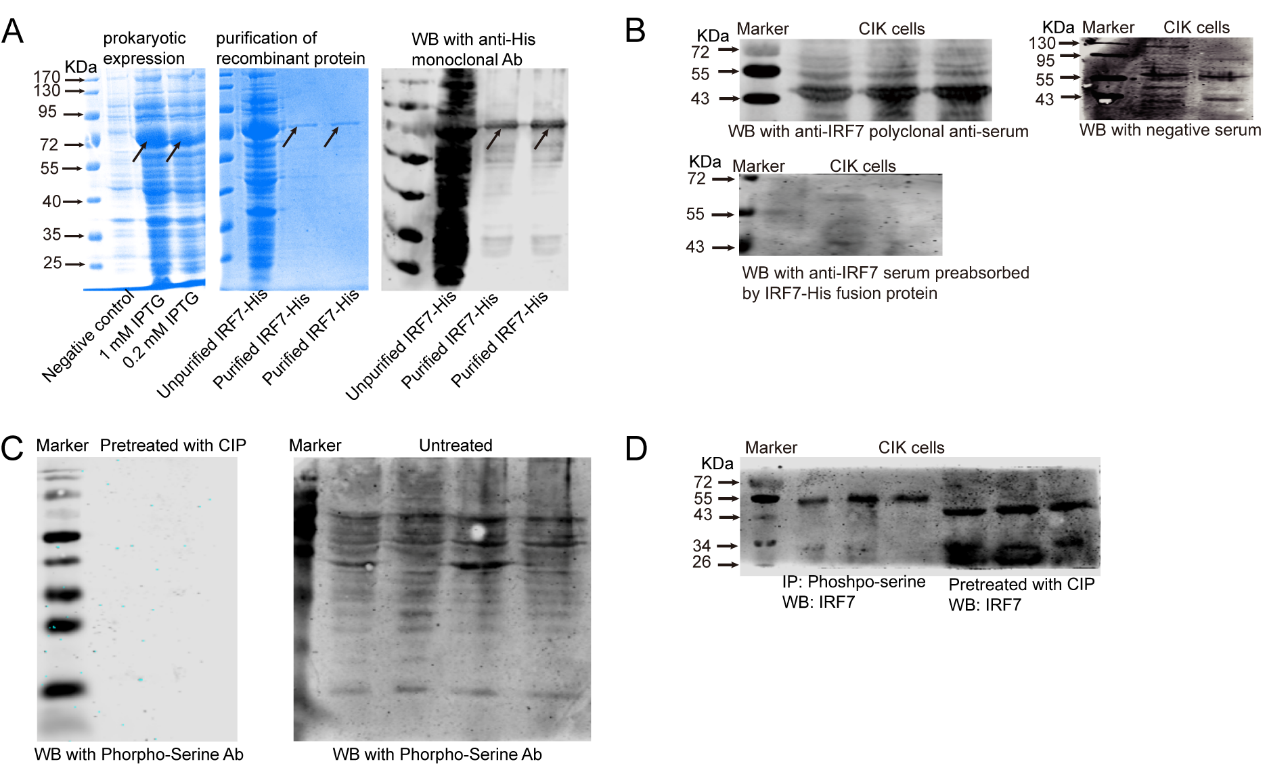


**Supplemental FIGURE 4** **Preparation of rabbit anti-IRF7 polyclonal anti-serum and verification of the phosphorylation form of CiIRF7**. **(A)** The full-length coding sequence of CiIRF7 was expressed as His-tagged fusion protein (CiIRF7-His) in BL21(DE3)pLysS *E. coli* strain. The lysates of non-induced bacteria, *i.e.*, Negative control, 1 mM or 0.2 mM IPTG induced bacteria, and the purified protein were separated in 12 % SDS-PAGE gels and stained with Coomassie brilliant blue R-250. Thereafter, the unpurified CiIRF7-His and purified CiIRF7-His were further confirmed by Western blot analysis with anti-His monoclonal antibody. Slanted arrows point to the target bands. **(B)** CIK cells were seeded into a 6-well plates, and lysed by NP-40 lysis buffer 24 h latter. The cytolysates were separated in 12 % SDS-PAGE gels, and then transblotted onto a NC membrane for Western blotting analysis. The anti-IRF7 antiserum from the immunized rabbit was able to recognize a cellular protein with a molecular mass of approximate 49 KDa, while the negative serum can recognize sundry proteins but not the expected size protein, and by using anti-IRF7 antiserum pre-absorbed with fusion protein CiIRF7-His, the expected protein band was not detected. **(C)** CIK cells cultured in a 10 cm plate were lysed by NP-40 lysis buffer supplemented with 1 mM PMSF, serine/threonine phosphatase inhibitor, tyrosine phosphatase inhibitor, and protease inhibitor cocktails, and then the co-immunoprecipitation and Western blot were performed by the phospho-serine antibody. No protein was detected in the cell lysate treated with CIP, but numerous proteins were detected in the untreated cell lysate, indicating the CIP and phospho-serine antibody can be used for the subsequent experiments. **(D)** Samples were prepared according to above described procedures. A portion of protein samples were used for immunoprecipitation, while other samples were treated with CIP. The bands of phosphorylation-formed CiIRF7 is larger than those of unphosphorylated CiIRF7, and they do not exist in the CIP treated samples.


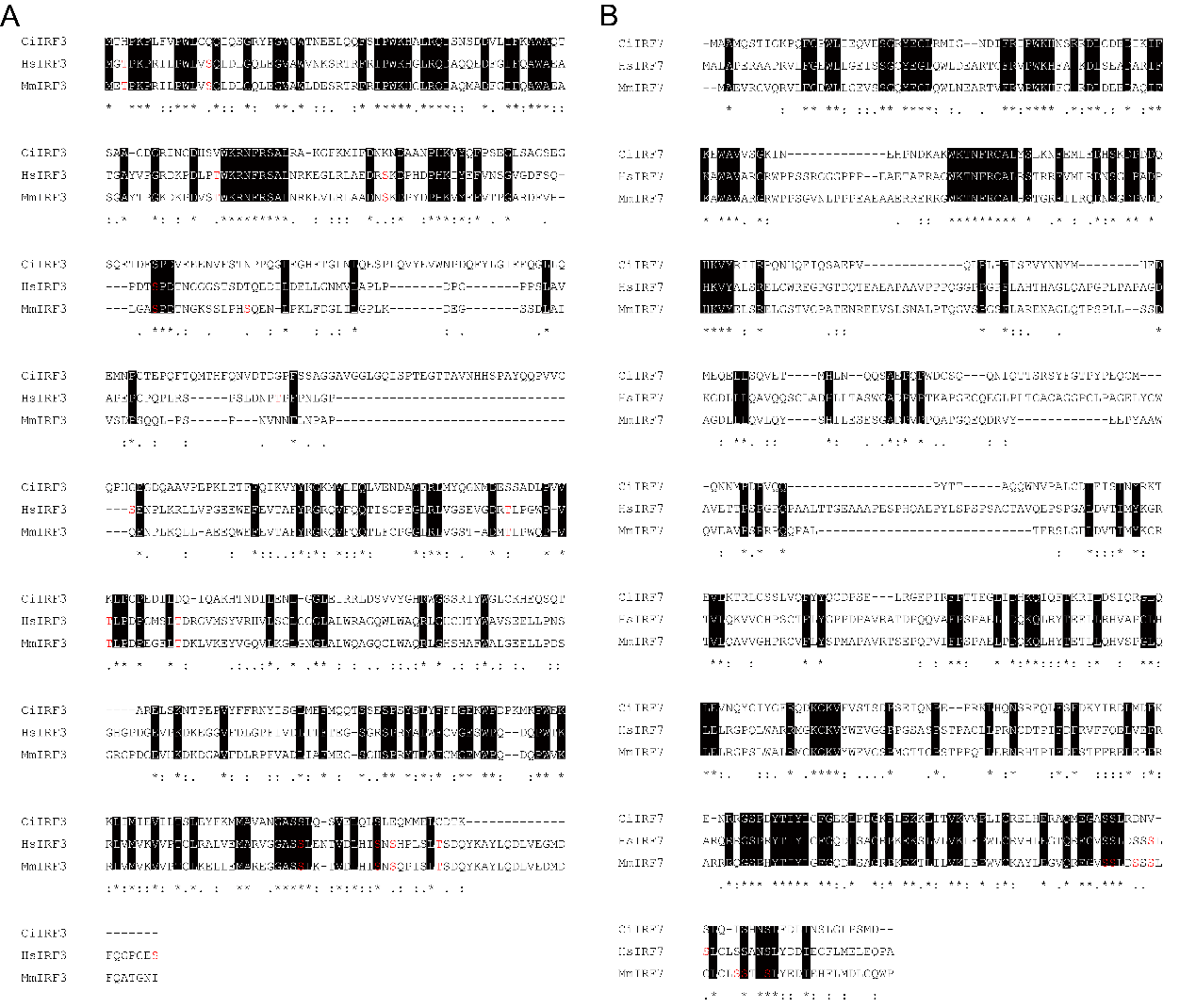


**Supplemental FIGURE 5 Multiple alignment of mature peptide sequences of IRF3 and IRF7 from grass carp, human and mouse**. Sequence alignments were obtained using CLUSTAL O program (V1.2.4) (<http://www.ebi.ac.uk/Tools/msa/clustalo/>), and the conserved residues are shaded. According to previous results (65-67), identified phosphorylation residues of human and mouse IRF3 (HsIRF3 and MmIRF3) **(A)** or IRF7 (HsIRF7 and MmIRF7) **(B)** are indicated by red fonts. Identical residues are indicated by asterisks, whereas those with high or low similarity are indicated by a colon (:) and period (.), respectively.

65. Gu L, Fullam A, Brennan R, Schroder M. Human DEAD box helicase 3 couples IkappaB kinase epsilon to interferon regulatory factor 3 activation. *Mol Cell Biol* (2013) 33(10): 2004-15. doi: 10.1128/MCB.01603-12.

66. Shu C, Sankaran B, Chaton CT, Herr AB, Mishra A, Peng J, et al. Structural insights into the functions of TBK1 in innate antimicrobial immunity. *Structure* (2013) 21(7): 1137-48. doi: 10.1016/j.str.2013.04.025.

67. Caillaud A, Hovanessian AG, Levy DE, Marie IJ. Regulatory serine residues mediate phosphorylation-dependent and phosphorylation-independent activation of interferon regulatory factor 7. *J Biol Chem* (2005) 280(18): 17671-7. doi: 10.1074/jbc.M411389200.
